# Global and Regional Risk Factors for Vision Impairment in 2020 and Multi-decadal Trends 1990–2020: A Meta-Analysis and Visual Display Based on Prevalence Data From the Global Burden of Disease study and Vision Loss Expert Group

**DOI:** 10.1007/s44402-026-00208-y

**Published:** 2026-10-16

**Authors:** Michele Bottone, Jost Jonas, Jaimie Steinmetz, Tasanee Braithwaite, Robert Casson, Joshua Ehrlich, Hugh R. Taylor, David Friedman, Nathan Congdon, John Kempen, Janet Leasher, Ningli Wang, Rohit Khanna, Tunde Peto, Joao M. Furtado, Tien Y. Wong, Mary E. Hartnett, Maria V. Cicinelli, Nina Tahhan, Van C. Lansingh, Sophia Y. Wang, Ian Tapply, Paul Briant, Theo Vos, Serge Resnikoff, Seth R. Flaxman, Rupert R. A. Bourne

**Affiliations:** 1 Imperial College, London, UK; 2 Rothschild Foundation Hospital, Paris, France; 3 Institute for Health Metrics and Evaluation, Seattle, Washington USA; 4 Centre for Translational Medicine, King’s Health Partners, London, UK; 5 University of Adelaide, Adelaide, South Australia Australia; 6 University of Michigan, Ann Arbor, Michigan USA; 7 University of Melbourne, Melbourne, Victoria Australia; 8 Massachusetts Eye and Ear Infirmary, Boston, Massachusetts USA; 9 Queen’s University Belfast, Belfast, UK; 10 Nova Southeastern University, Davie, Florida USA; 11 Beijing Tongren Hospital, Beijing, China; 12 L V Prasad Eye Institute, Hyderabad, India; 13 Universidade de Sao Paulo, Sao Paulo, Brazil; 14 Tsinghua University, Beijing, China; 15 Stanford University, Stanford, California USA; 16 Vita-Salute San Raffaele University, Milan, Italy; 17 University of New South Wales, Sydney, New South Wales Australia; 18 University of Miami, Miami, Florida USA; 19 Cambridge University Hospitals NHS Foundation Trust, Cambridge, UK; 20 University of Arizona, Tucson, Arizona USA; 21 University of Oxford, Oxford, UK; 22 Anglia Ruskin University, Cambridge, UK; 23 The Fred Hollows Foundation, Sydney, New South Wales Australia; 24 Visibility Metrics LLC, New York, New York USA; 25 Ophthalmology Unit, IRCCS San Raffaele Scientific Institute, Milan, Italy; 26 Eye Repair Unit, Division of Neuroscience, IRCCS San Raffaele Scientific Institute, Milan, Italy; 27 Ufa Eye Research Institute, Ufa, Russia; 28 Vision and Eye Research Institute, School of Medicine, Anglia Ruskin University, Cambridge, UK; 29 The Retina Clinic, London, UK; 30 University Hospital, Dijon, France; 31 International Centre for Eye Health, London School of Hygiene & Tropical Medicine, London, UK; 32 School of Optometry and Vision Science, University of Auckland, Auckland, New Zealand; 33 University of Malta, Msida, Malta; 34 Kings College, London, UK; 35 Department of Ophthalmology, Centre for Innovation & Precision Eye Health, Yong Loo Lin School of Medicine, National University of Singapore, Singapore, Singapore; 36 Singapore Eye Research Institute, Singapore National Eye Centre, Singapore, Singapore; 37 School of Medicine, Vita-Salute San Raffaele University, Milan, Italy; 38 Department of Ophthalmology, IRCCS San Raffaele Scientific Institute, Milan, Italy; 39 Orbis International, New York, New York USA; 40 Zhongshan Ophthalmic Center, Sun-Yat-sen University, Guangzhou, China; 41 Massachusetts Eye & Ear Infirmary, Harvard Medical School, Boston, Massachusetts USA; 42 Department of Surgery (Ophthalmology), University Tunku Abdul Rahman, Petaling Jaya, Malaysia; 43 Duke-NUS Medical School, National University of Singapore, Singapore, Singapore; 44 Centre for Eye Research Australia, Royal Victorian Eye and Ear Hospital, East Melbourne, Victoria Australia; 45 University of Alabama, Birmingham, Alabama USA; 46 Department of Ophthalmology and Visual Sciences, University of Michigan, Ann Arbor, Michigan USA; 47 Institute for Social Research, University of Michigan, Ann Arbor, Michigan USA; 48 National Eye Institute, Bethesda, Maryland USA; 49 Department of Ophthalmology and Visual Sciences, Federal University of Sao Paulo, Sao Paulo, Brazil; 50 Department of Computer Science, University of Oxford, Oxford, UK; 51 Ribeirão Preto Medical School, University of São Paulo, Ribeirão Preto, Brazil; 52 Institute of Ophthalmology, London, UK; 53 Medical Research Foundation, Chennai, India; 54 University of Georgia, Athens, Georgia USA; 55 Byers Eye Institute, Stanford University, Palo Alto, California USA; 56 Alberta Children’s Hospital, Calgary, Alberta Canada; 57 Privatpraxis Prof. Jonas und Dr. Panda-Jonas, Heidelberg, Germany; 58 Beijing Visual Science and Translational Eye Research Institute (BERI), Beijing Tsinghua Changgung Hospital, Tsinghua Medicine, Tsinghua University, Beijing, China; 59 New York Eye and Ear Infirmary of Mount Sinai, Icahn School of Medicine at Mount Sinai, New York, New York USA; 60 Department of Ophthalmology, University Hospital Heidelberg, Heidelberg, Germany; 61 Associated Ophthalmologists of Monastir, Monastir, Tunisia; 62 Department of Ophthalmology, Mass Eye and Ear/Schepens Eye Research Institute, Boston, Massachusetts USA; 63 Departments of Ophthalmology and Global Health & Social Medicine, Harvard Medical School, Boston, Massachusetts USA; 64 MyungSung Christian Medical Center (MCM) Eye Unit and MyungSung Medical College, Addis Ababa, Ethiopia; 65 Department of Ophthalmology, Addis Ababa University School of Medicine, Addis Ababa, Ethiopia; 66 University Hospital of Monastir, Monastir, Tunisia; 67 UT Southwestern Medical Center, Dallas, Texas USA; 68 HelpMeSee, Jersey City, New Jersey USA; 69 Department of Public Health Sciences, University of Miami, Miami, Florida USA; 70 Department of Ophthalmology and Visual Sciences, John A. Moran Eye Center, University of Utah, Salt Lake City, Utah USA; 71 Mexican Institute of Ophthalmology, Queretaro, Mexico; 72 Camden Family Health, Summersville, West Virginia USA; 73 University of Poitiers, Poitiers, France; 74 University of Illinois, Urbana, Illinois USA; 75 Centre for Optometry & Vision Science Research, Ulster University, Belfast, UK; 76 Prof. Brien Holden Eye Research Centre, Gullapalli Pratibha Rao International Centre for Advancement of Rural Eyecare, L V Prasad Eye Institute, Hyderabad, India; 77 Jewish Guild Healthcare, New York, New York USA; 78 University of KwaZulu-Natal, Durban, South Africa; 79 Suraj Eye Institute, Nagpur, India; 80 University of Social Science, Lodz, Poland; 81 University Vita Salute, Ospedale San Raffaele, Milan, Italy; 82 School of Optometry and Vision Science, University of New South Wales, Sydney, New South Wales Australia; 83 Wilmer Eye Institute, Johns Hopkins University, Baltimore, Maryland USA; 84 Johns Hopkins University, Baltimore, Maryland USA; 85 University of Milan, Milan, Italy; 86 Mt Sinai School of Medicine, New York, New York USA; 87 University of Washington, Seattle, Washington USA; 88 Department of Ophthalmology, Faculty of Medicine, University of Indonesia, Depok, Indonesia; 89 University of Pennsylvania, Philadelphia, Pennsylvania USA; 90 Melbourne School of Population and Global Health, University of Melbourne, Melbourne, Victoria Australia; 91 University of Thessaloniki, Thessaloniki, Greece; 92 University of Crete Medical School, Heraklion, Greece; 93 Southern California Eye Institute, Los Angeles, California USA; 94 University of Florence, Florence, Italy; 95 Capital Medical University, Beijing, China; 96 Beijing Institute of Ophthalmology, Beijing, China; 97 Johns Hopkins Hospital, Baltimore, Maryland USA; 98 Tsinghua Medicine, Tsinghua University, Beijing, China; 99 Singapore National Eye Center, Singapore, Singapore

**Keywords:** Blindness, Epidemiology, Pollution, Risk factors, Smoking, Vision impairment

## Abstract

**Purpose:**

To investigate global and regional risk factors for blindness and vision impairment pertaining to all geographical locations between 1990 and 2020.

**Methods:**

(1) Twenty-four covariates were selected with a potential link to vision impairment from the Global Burden of Disease Study (GBD) Covariates database as predictive factors. (2) From these, the linear association was investigated, with crude and age-standardised prevalence estimates of blindness and distance and near vision impairment in 2020 by using data for the whole population, sex and 5-year age groups, and by stratifying severity through mild, moderate, severe and total impairment. (3) Analysis investigated whether percentage rates of change in risk factors were associated with rates of change in prevalence in 1990–2020.

**Results:**

For adults (50+ years), for crude and age-standardised vision loss prevalence estimates for the year 2020, a significant negative association of smoking prevalence was found initially for all persons and for each sex, which appears to be due to ecological confounding and became a positive association using more granular regional and age group data. Maternal care and immunisation, sanitation proportion, Health Access and Quality Index and Socio-demographic Index were other significant age-standardised negative associations. Ophthalmologists/million population had a weak but significant protective effect, and positive associations were found for pollution (outdoor and indoor), stronger for greater vision impairment severities. Years in education per capita were weakly, negatively associated with vision loss prevalence, yet zero education proportion had a stronger but positive association for both sexes.

**Conclusions:**

This study confirmed associations at population level globally, which appear to be stable over decades. In addition, the study quantified the contribution of epidemiologically-based risk factors to vision loss at a GBD super-regional level, which is novel. This opens useful avenues for targeted intervention by policymakers. For example, reduction in sex inequity in blindness rates over 30 years has been positively associated with reducing overall prevalence of blindness.

Key Points

- Risk factors for vision loss such as female sex, less education or more inequality in education and less access to healthcare were confirmed at the population level both globally and by Global Burden of Disease super-region and appear to be stable over decades.
- Indoor and outdoor pollution were associated with vision impairment, with a stronger association at more severe levels of vision loss. The association between second-hand smoke and vision impairment was stronger in older age in women. Obesity was protective for vision loss, save for Sub-Saharan Africa, where a higher obesity prevalence was associated with greater VI prevalence.
- Behavioural factors such as smoking and alcohol consumption were protective at the global level because of confounding, yet when considering regions with more prevalent smoking or alcohol consumption such as South East Asia, East Asia and Oceania and Latin America and the Caribbean, greater reductions in these risk factors in 1990–2020 were associated with greater reductions in blindness prevalence, while increases in these risk factors were associated with lesser reductions.

## Introduction

Over the last three decades, momentous changes have occurred at a global and regional level in the burden of vision impairment (VI). In 1999, the World Health Organization and the International Agency for the Prevention of Blindness launched the ‘VISION2020: the Right to Sight’ campaign with strategic blindness and VI initiatives. Contributing to this effort, the Vision Loss Expert Group (VLEG) and the Global Burden of Disease (GBD) Study have collaboratively updated all-causes and cause-specific prevalence estimates, investigated geographical and age- and sex-specific changes from 1990 to 2020 with a systematic analysis of new population-based survey data from 1980 to 2018, modelling vision loss prevalence estimates and forecasts through 2050 [1, 2].

While the age-standardised prevalence of VI and blindness has been declining [1], close inspection of the updated data reveals a richer picture, globally and at a regional and country level, and important questions remain to be answered. A key consideration is the relative contribution of risk factor (both modifiable and non-modifiable) trends to this decline, which have not been fully investigated, in spite of being of great importance for public health policy and clinical interventions. Overall, despite a decrease in the age-standardised prevalence of VI and blindness, the number of VI or blind individuals has increased globally, and it is unclear if this is reflective of ageing and population growth or an increase in the prevalence of chronic disease and other risk factors related to VI [3, 4].

In this regard, complex eye-related diseases leading to VI have a stratified variety of determinants due to their aetiology and the interplay of risk factors such as age, sex, ethnicity, concurrent systemic diseases (e.g., diabetes, hypertension), modifiable behaviours (e.g., smoking), regional location, socio-economic status, healthcare access, genetic susceptibility, immediate and lifecycle environments, all of which are associated with vision loss and its effect on the individual´s productivity and quality of life. For example, reduced vision can lead to lower wellbeing, a higher incidence of falls in older persons [5–7] and may contribute to cognitive dysfunction [8, 9]. Disentangling the myriad causal effects of potential factors can be challenging [10], and consequently, analyses of risk factors for vision loss have involved longitudinal analyses or self-reported surveys, usually in single specific populations for specific geographical locations.Population-level associations between risk factors and vision loss have been considered in specific countries, across high-income and low-income countries [11, 12] and for specific severities of VI [13], yet few studies have addressed global, regional and cross-country factors.

To address these important gaps, the present study expanded the focus of the GBD 2019 prevalence data [1, 2] to account for global and regional risk factors. Demographic, socio-economic, behavioural, physiological and environmental risk factors for vision loss are investigated using the updated estimates and models reported in the GBD 2019 [1], including analysis of temporal trends in sex ratios and examining the contribution of the overall change due to the risk factors.

## Methods

### Data Sources

The Institute for Health Metrics and Evaluation and the VLEG produce updated prevalence estimates for all countries, 21 regions and seven GBD super-regions, recorded at 5-year time intervals [1].

A database of covariates was preliminarily screened across GBD modelling (https://ghdx.healthdata.org/record/ihme-data/gbd-2023-covariates-1980-2023) for those with a hypothesised link to eye disease. After considering the available literature (see Appendix Text S1), the research team selected 24 covariates potentially related to VI, stratified by age and sex. Overall, the potential population risk factors for VI considered in this work included a mixture of covariates, ranging from common behavioural factors (e.g., smoking, alcohol consumption, nutrition), physiological markers, proxies for educational attainment quality and access indices for healthcare, environmental risk factors and socio-demographic and inequality indices. These were supplemented with country estimates for the number of ophthalmologists per million population as a proxy for access to eye care [14]. Almost all of these covariates came from repeated monitoring and modelling of surveys and provided an opportunity to associate the variability of the VI and disease burden trends with the corresponding risk factor trends in the whole population.

### Definitions and Statistical Methods

The association of potential risk factors were analysed, with crude and age-standardised estimates of prevalence of vision loss for all-causes combined in 2020 (forecast from GBD 2019) for the following severity levels: blindness (BI), moderate and severe vision impairment (MSVI), mild VI and near VI from uncorrected presbyopia (near vision impairment (NVI)). Following the WHO, the International Classification of Diseases 11th edition criteria for vision loss was used [15], which categorises people according to presenting vision in the better eye (Appendix Text S2).

Additionally, the trend in percentage rates of change in risk factors to the rates of change of prevalence between 1990 and 2020 were analysed.

For the changes in each covariate/ group of covariates, the global mean associations and their 95% uncertainty intervals (UIs) were estimated using posterior draws under a generalised linear model, examining both unweighted regressions and coefficient values weighted by country population size. In addition to global estimates, associations for the seven GBD super-regions of (i) high income (Australasia, Western Europe, Asia-Pacific and North America); (ii) Latin America and Caribbean; (iii) Sub-Saharan Africa; (iv) South Asia; (v) South East Asia, East Asia and Oceania; (vi) North Africa and Middle East and (vii) Central Europe, Eastern Europe and Central Asia were also calculated.

For all associations computed, the outcome variable was one of the four VI severity prevalences in 2020 using GBD 2019 estimates and its relative percentage rate of change between 1990 and 2020, versus the covariate (or its rate of change from 1990) measured in that year or, where missing, using the last available estimate brought forward. Three models were fitted, specified as a generalised linear model of: (a) covariates available for all persons, (b) women and men separately and for the age-specific estimates or (c) as a model specified at the single level of men and women and at the further level of 5-year age cohorts. The multivariable analysis considered the joint effect of all factors in the model, compared to a simple univariable analysis of a single factor. Regression coefficients are interpreted such that for each change in either units or proportions of the covariate, under the assumptions of the statistical model, one can expect a given (positive or negative) percentage change in prevalence. This allows a direct interpretation in terms of odds ratios (ORs) for their effect. For example, a 0.98 OR corresponds to a 2% reduction in risk (odds), compared to no impairment (a 0.02 coefficient in the regression; a protective factor). For risk factors, one would expect an OR > 1 in the relationship between rates of change, meaning that any reduction in VI prevalence over 1990–2020 can be ‘explained’ in the model by a reduction in the covariate and vice versa, i.e., any increase in VI prevalence (or lesser reduction) can be explained by an increase (or lesser reduction) in harmful risk factors.

All statistical analyses were performed in R version 3.6.3 (r-project.org) using the *stan_glm* function (*stan_glmer* for the two-level age-specific hierarchical model) in the *rstanarm* package with the default of Gaussian priors on the intercept and coefficient and an exponential distribution for sigma. Further details on statistical methods are given in the Appendix Text S2.

## Results

**Fig. 1:**
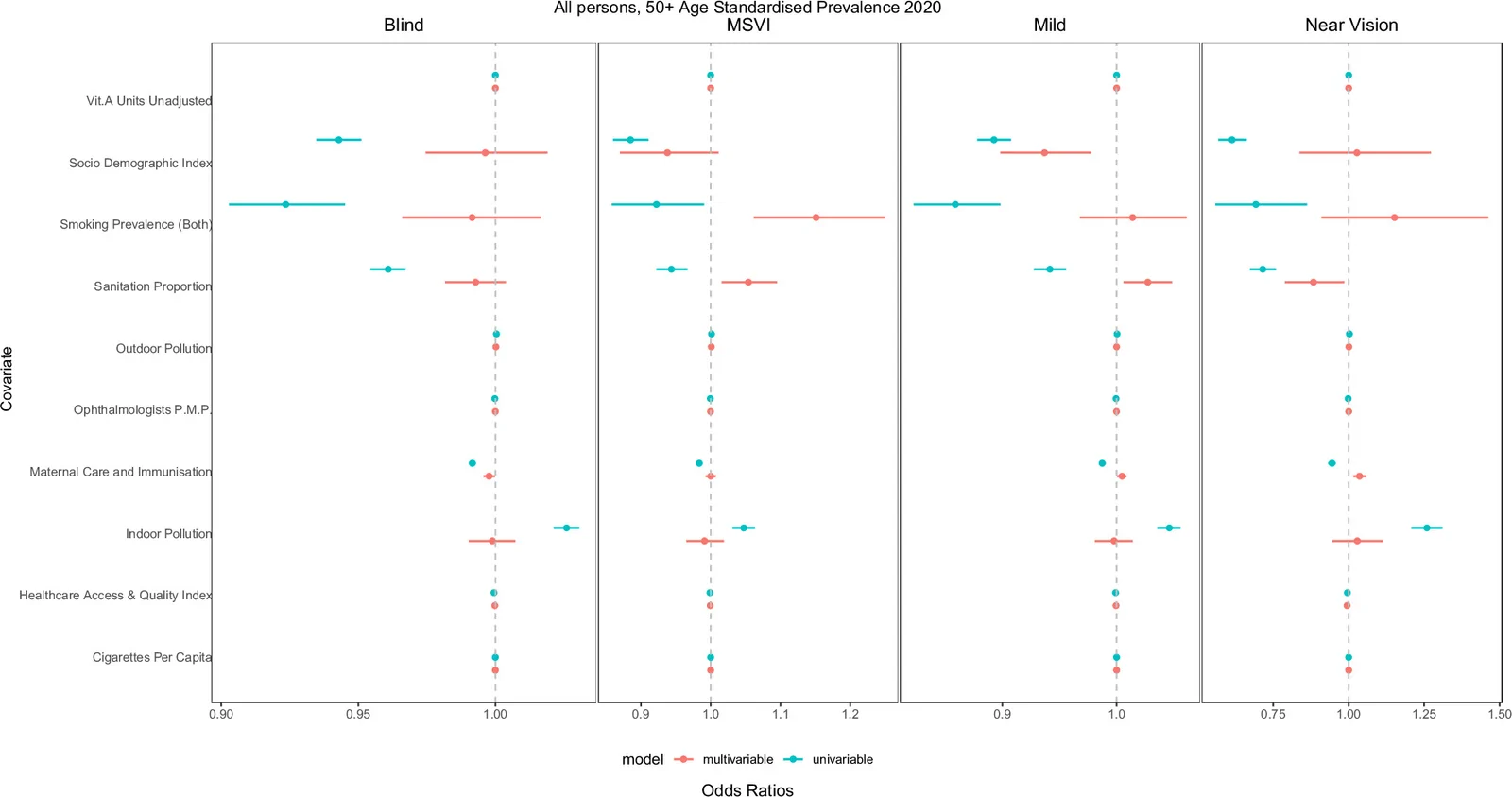
Global Risk Factors associated with age-standardised prevalence of blindness, moderate and severe (MSVI), Mild and Near Vision impairments for 193 countries: Single and multiple variable odds ratios and 95% UI for *All Persons*. P.M.P = Per Million Population.

**Fig. 2:**
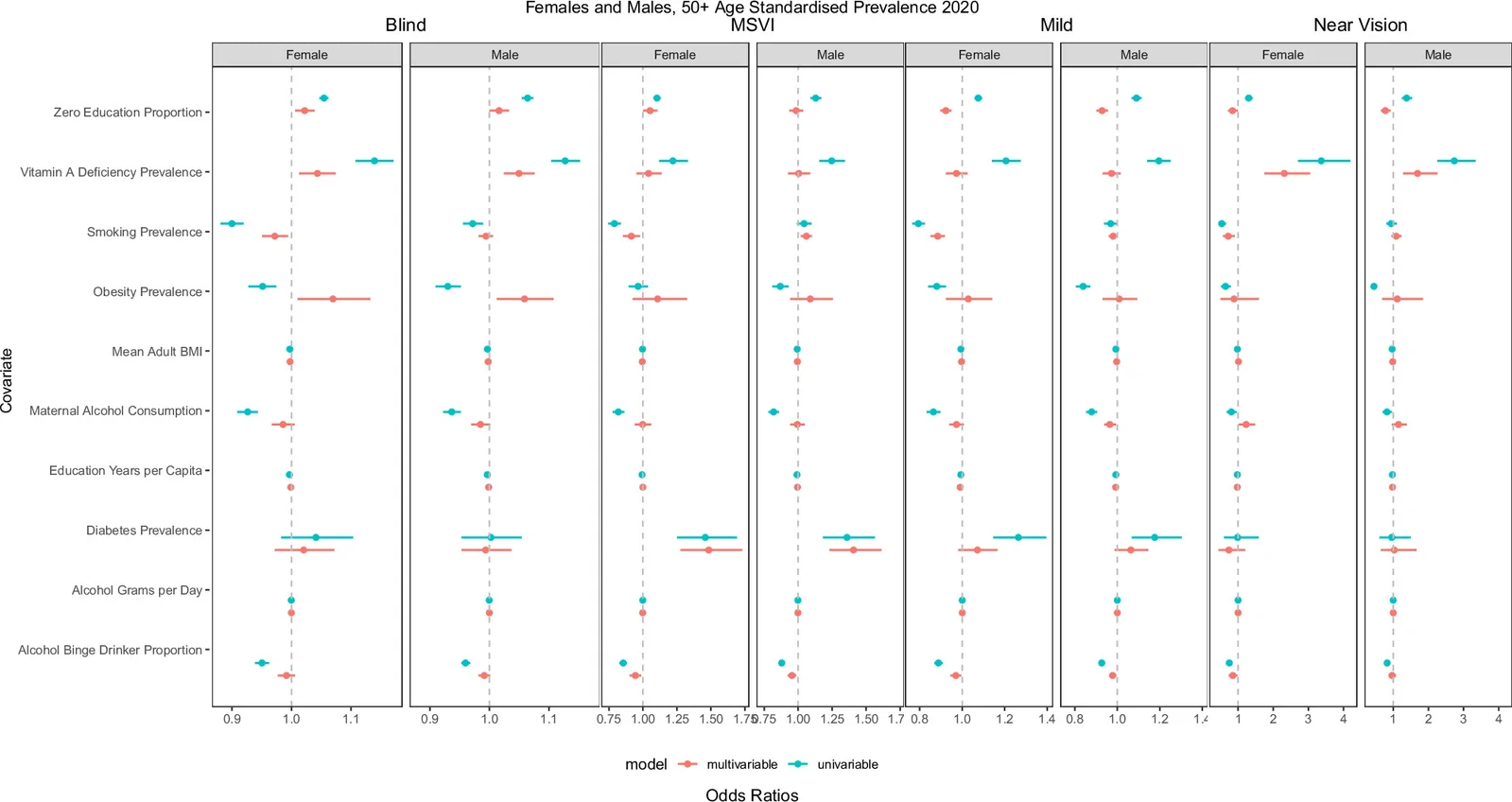
Global Risk Factors associated with age-standardised prevalence of blindness, moderate and severe (MSVI), Mild and Near Vision impairments for 193 countries: Single and multiple variable odds ratios and 95% UI for *Females and Males*.

**Fig. 3:**
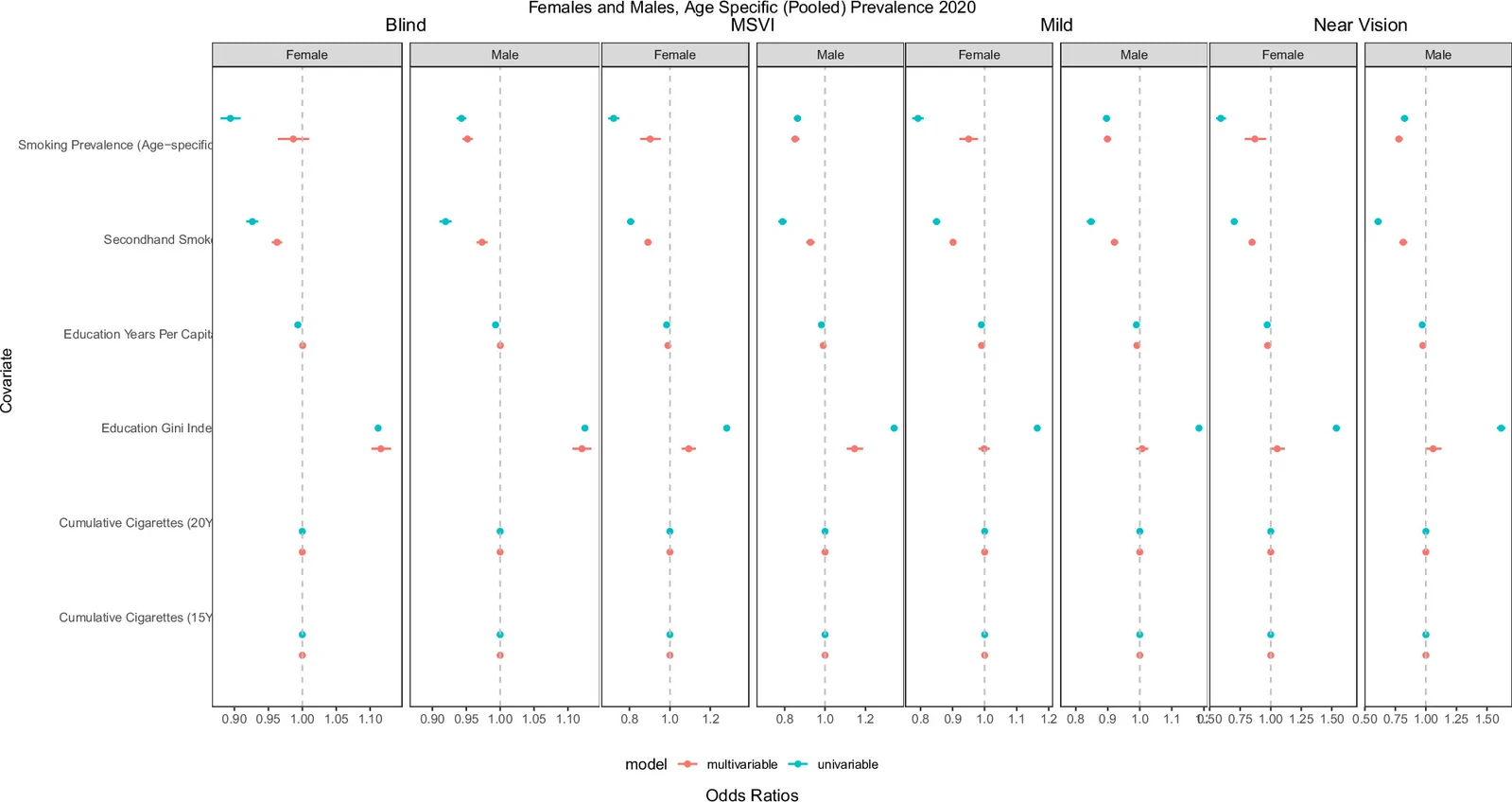
Global Risk Factors associated with age-standardised prevalence of blindness, moderate and severe (MSVI), Mild and Near Vision impairments for 193 countries: Single and multiple variable odds ratios and 95% UI for *Age Specific Pooled*.

Population-weighted and unweighted global coefficient estimates for risk factors did not differ significantly from each other for the global associations. Consequently, in all tables and figures that follow, global coefficients and ORs refer to unweighted estimates for all 193 countries, labelled as ‘World’ in the figures of the Appendix (Supplementary Material). Global risk factors associated with age-standardised prevalence of blindness, MSVI, mild VI and NVIs for 193 countries are presented with single- and multiple variable ORs in Fig. 1 (for all persons), Fig. 2 (for females and males) and Fig. 3 (age-specific model), and in tabulated form in Appendix Tables S1–S3. The statistically significant associations between risk factors and blindness are presented in Table 1 (for MSVI: see Appendix Table S4), and significant risk factors for percentage rates of change between 1990 and 2020 in prevalence of blindness and MSVI are shown in Table 2. The results of the multiple variable analysis generally mirrored in sign those of the univariable analysis (it was noted that their relative significance was only valid in the context of the model specification).

**Table 1:** Significant risk factors associated with age-standardised prevalence of blindness for 193 countries: single and multiple variable analysis, global estimated odds ratios [see Appendix Table S4 for moderate and severe vision impairment].

Prevalence	Blind, single variable		Blind, multiple variable	
Covariate, all persons	OR (95% UI)		OR (95% UI)	
Smoking (all)	0.9235 (0.9028, 0.9452)		0.9915 (0.966, 1.0166)	
Outdoor pollution	1.0004 (1.0003, 1.0005)		1.0002 (1.0001, 1.0003)	
Indoor pollution	1.026 (1.0213, 1.0306)		0.9989 (0.9903, 1.0073)	
Sanitation (proportion)	0.9609 (0.9544, 0.9672)		0.9928 (0.9817, 1.0038)	
Maternal care and immunisation	0.9916 (0.9904, 0.9928)		0.9977 (0.9956, 0.9998)	
Healthcare Access and Quality Index	0.9995 (0.9995, 0.9996)		0.9998 (0.9997, 1)	
Ophthalmologists PMP	0.9998 (0.9997, 0.9998)		1 (0.9999, 1)	
Socio-demographic Index	0.9429 (0.9347, 0.9511)		0.9963 (0.9746, 1.019)	
**Prevalence**	**OR (95% UI)**		**OR (95% UI)**	
**Covariate, by sex**	**Female**	**Male**	**Female**	**Male**
Alcohol binge drinker (proportion)	0.9504 (0.9386, 0.9621)	0.9599 (0.9528, 0.9677)	0.9916 (0.9768, 1.0063)	0.9913 (0.9815, 1.0013)
Maternal alcohol consumption	0.9264 (0.9088, 0.9435)	0.9367 (0.9223, 0.9518)	0.9858 (0.9671, 1.0053)	0.9849 (0.9693, 1.0011)
Vitamin A deficiency	1.1395 (1.1077, 1.1715)	1.1274 (1.104, 1.1525)	1.0436 (1.0129, 1.0743)	1.0497 (1.0245, 1.0759)
Education (years per capita)	0.9965 (0.9961, 0.9970)	0.9965 (0.9960, 0.9970)	0.9988 (0.9975, 1.0001)	0.9988 (0.9978, 0.9998)
Zero education (proportion)	1.0547 (1.047, 1.0621)	1.0641 (1.0544, 1.0739)	1.0222 (1.006, 1.0389)	1.0162 (1.0001, 1.0331)
Smoking (F/M)	0.8999 (0.8809, 0.9197)	0.9719 (0.9558, 0.9894)	0.9719 (0.9506, 0.9943)	0.9942 (0.9819, 1.0061)
Second-hand smoke (age-specific, pooled)	0.9264 (0.9175, 0.9350)	0.9195 (0.9106, 0.9283)	0.9628 (0.9552, 0.9702)	0.9732 (0.9652, 0.9813)
Education Gini (age-specific, pooled)	1.1119 (1.1073, 1.1165)	1.1250 (1.1202, 1.1298)	1.1159 (1.1020, 1.1309)	1.1206 (1.1065, 1.1346)

*UI* uncertainty interval.

**Table 2:** Significant risk factors for % rates of change between 1990 and 2020 in prevalence of blindness, moderate and severe vision impairment (MSVI) for 193 countries: single and multiple variable analysis, global estimated odds ratios.

Rates of change, 1990 to 2020, univariable	Blindness		MSVI	
% Change, all persons	% change OR 1990–2020 (95% UI)		% change OR 1990–2020 (95% UI)	
Smoking (all)	0.9212 (0.8558, 0.9944)		0.9803 (0.9547, 1.0067)	
Outdoor pollution	0.7669 (0.6891, 0.8543)		0.9257 (0.8892, 0.9619)	
Sanitation proportion	0.9891 (0.9805, 0.9975)		0.9963 (0.9935, 0.9992)	
Healthcare Access and Quality Index	0.8894 (0.8642, 0.9161)		0.9806 (0.9697, 0.9916)	
Socio-demographic Index	0.8869 (0.8557, 0.9192)		0.9725 (0.9591, 0.9860)	
**% Change, by sex**	**% Change OR 1990–2020 (95% UI)**		**% Change OR 1990–2020 (95% UI)**	
	**Female**	**Male**	**Female**	**Male**
Obesity	0.9783 (0.9701, 0.9866)	0.9807 (0.9750, 0.9864)	0.9974 (0.9943, 1.0005)	0.9981 (0.9959, 1.0004)
Mean BMI	0.3615 (0.2410, 0.5533)	0.2630 (0.1615, 0.4311)	0.8838 (0.7582, 1.0291)	0.9172 (0.7604, 1.0982)
Vitamin A deficiency	1.3728 (1.2589, 1.5010)	1.4368 (1.3208, 1.5597)	1.0421 (1.0070, 1.0793)	1.0518 (1.0166, 1.0888)
Education (years per capita)	0.9688 (0.9549, 0.9823)	0.9293 (0.9007, 0.9589)	0.9870 (0.9820, 0.9920)	0.9782 (0.9676, 0.9892)
Smoking (F/M)	0.9698 (0.9017, 1.0426)	0.9094 (0.8465, 0.9809)	0.9889 (0.9639, 1.0151)	0.9771 (0.9508, 1.0046)
Second-hand smoke (age-specific, pooled)	0.8931 (0.8707, 0.9166)	0.8770 (0.8492, 0.9062)	0.9366 (0.9288, 0.9448)	0.9658 (0.9556, 0.9761)
Education Gini (age-specific, pooled)	1.0520 (1.0315, 1.0729)	1.1595 (1.1314, 1.1889)	0.9934 (0.9867, 1.0001)	1.0133 (1.0057, 1.0216)
**Rates of change, 1990 to 2020, multivariable**	**Blindness**		**MSVI**	
**% Change, all persons**	**% change OR 1990–2020 (95% UI)**		**% change OR 1990–2020 (95% UI)**	
Smoking (all)	0.9414 (0.8533, 1.0404)		0.9894 (0.9472, 1.0346)	
Outdoor pollution	0.8726 (0.7841, 0.9700)		0.9500 (0.9064, 0.9941)	
Sanitation proportion	0.9907 (0.9833, 0.9983)		0.9973 (0.9939, 1.0008)	
Healthcare Access and Quality Index	0.9021 (0.8664, 0.9406)		0.9915 (0.9741, 1.0086)	
Socio-demographic Index	0.9885 (0.9301, 1.0511)		0.9887 (0.9632, 1.0147)	
**% Change, by sex**	**% change OR 1990–2020 (95% UI)**		**% change OR 1990–2020 (95% UI)**	
	**Female**	**Male**	**Female**	**Male**
Obesity	0.9919 (0.9820, 1.0015)	0.9922 (0.9862, 0.9982)	1.0010 (0.9973, 1.0048)	1.0008 (0.9983, 1.0035)
Mean BMI	0.6238 (0.3838, 1.0118)	0.5171 (0.3101, 0.8518)	0.9025 (0.7397, 1.1007)	0.9418 (0.7556, 1.1807)
Vitamin A deficiency	1.2128 (1.1005, 1.3352)	1.2502 (1.1442, 1.3681)	1.0095 (0.9704, 1.0505)	1.0267 (0.9864, 1.0703)
Education (years per capita)	0.9671 (0.9523, 0.9828)	0.9432 (0.9161, 0.9713)	0.9866 (0.9802, 0.9928)	0.9778 (0.9651, 0.9904)
Smoking (F/M)	1.0429 (0.9785, 1.1133)	0.9883 (0.9300, 1.0528)	1.0036 (0.9795, 1.0295)	0.9951 (0.9678, 1.0232)
Second-hand smoke (age-specific, pooled)	0.9760 (0.9497, 1.0036)	0.9636 (0.9331, 0.9954)	0.9556 (0.9470, 0.9645)	0.9897 (0.9789, 1.0003)
Education Gini (age-specific, pooled)	1.1076 (1.0869, 1.1287)	1.1508 (1.1241, 1.1791)	1.0032 (0.9965, 1.0099)	1.0127 (1.0045, 1.0208)

*UI* uncertainty interval.

The global associations are summarised as follows for the single-variable and multivariable analyses.

### Single-Variable Analysis

Negative associations of smoking prevalence were found for all vision loss severities (except for MSVI for males, which was positive), for 50+ years age-standardised prevalence estimates for the year 2020, for both sexes combined (all persons model) and for each sex. Maternal care and immunisation (MCI), sanitation proportion and Socio-demographic Index (SDI) were other significant age-standardised negative associations for the all-persons model. Healthcare Access and Quality Index (HAQI) and the number of ophthalmologists per million population (Table 1 and Fig. 1) had a weak but significant protective effect. Both indoor and outdoor pollution were associated with VI, with a stronger association for greater VI severities. For sex-disaggregated data, diabetes prevalence and Vitamin A deficiency were associated with more VI, while obesity prevalence, proportion of alcohol binge drinkers and maternal alcohol consumption were associated with less VI. For this group, the magnitude of effect was stronger for females and weaker for more severe VI. More years of education per capita was weakly associated with less VI prevalence across countries, and proportion of zero education was associated with more VI for both sexes.

All other covariates for prevalence in 2020 had coefficients that were either statistically significant but close to zero, meaning no clinically important association or had UIs that widely overlapped zero (not statistically significant).

Super-regional breakdown of single-variable associations for age-standardised blindness and MSVI impairment of older persons (>50 years) is presented in Figs. S1 and S2 for all persons and by sex, respectively.

### Multiple Variable Analysis

Slightly negative significant associations were found for smoking prevalence (except MSVI, which showed a positive effect for males; weak significance) and proportion having sanitation with blindness and NVI, as well as a positive association of proportion having sanitation with MSVI and mild VI only.

Among males and females, variables shared by both sexes and most severities were (with negative effects) proportion of alcohol binge drinking and zero education, as well as positive effects of diabetes (except for blind males) and obesity (blindness and MSVI but not mild VI and near VI). The prevalence of Vitamin A deficiency was more associated with blindness and MSVI in females and more strongly with NVI for both sexes.

### Risk Factors Associated With Blindness and Vision Impairment and Super-Regional Contributions for 1990–2020 Rates of Change

**Fig. 4:**
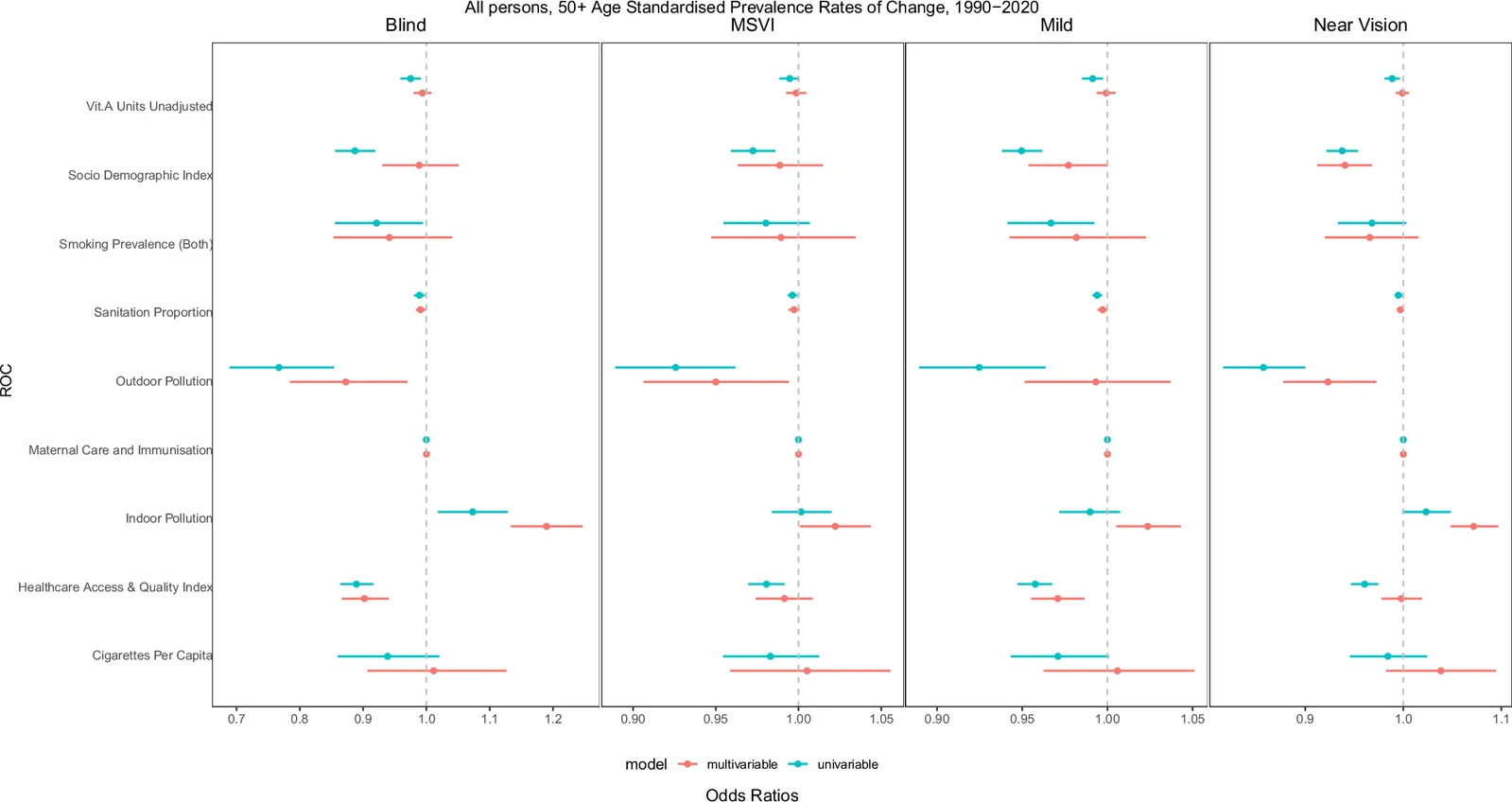
Global Risk Factors for % rates of change between 1990 and 2020 in prevalence of blindness, moderate and severe (MSVI), Mild and Near Vision impairments for 193 countries: Single and multiple variable odds ratios and 95% UI for *All Persons*.

Figure 4 presents the single- and multiple-variable risk factors for the relative percentage rate of change from 1990 to 2020 (in both prevalence and covariate) for all persons (see Appendix Fig. S3 for disaggregation by sex). The statistically significant associations between rates of change in risk factors and rates of change in blindness and MSVI are presented in Table 2.

Super-regional breakdowns of single-variable associations for percentage rate of change in age-standardised blindness and MSVI impairment of older persons (>50 years) are presented in Figs. S4 and S5 for all persons and by sex, respectively.

There was a significant negative single variable association between the percentage rates of change from 1990 to 2020 of smoking prevalence, outdoor pollution, HAQI, SDI, sanitation and MCI with the percentage change in prevalences of blindness and MSVI, for all persons (Table 2). These were also significant for the multiple-variable analysis, as was indoor pollution for blindness. Other associations were not significant. For data disaggregated for males and females (Table 2, lower section), negative associations were found for the rates of change of education years per capita, adult BMI, obesity prevalence and diabetes prevalence (MSVI only). These effects were very small compared with the rates of change of mean BMI. For vitamin A deficiency and alcohol binge drinker proportion, the association and direction were positive, which meant that the reduction in VI prevalence could be explained by a reduction in vitamin A deficiency and binge drinking prevalence. Across severities, women had a weaker association for education years per capita and a stronger association for obesity (unadjusted for cardiovascular/cerebrovascular disease) compared to men. These findings are also presented in Appendix Figs. S4 and S5 with super-regional associations, and are consistent across regions.

The all-persons multiple regressions found significant, but weak, negative coefficients for smoking, HAQI and SDI. For women and men, there was also a significant positive association for vitamin A deficiency.

### Age-Specific Risk Factors and Sex Ratios

When restricting the regression to a single population level, in addition to the apparently protective effect of smoking that was stronger for women, second-hand smoke and education Gini coefficient [16] (which measures inequality in educational attainment (years of schooling) within a population, ranging from 0 (perfect equality) to 1 (perfect inequality)) had a significant negative and positive association, respectively. Education years per capita also had a significant, protective, weak but increasing-by-severity-of-VI association. The pooled multivariable analysis also found these associations to hold with the same sign for all severities, except for mild and near vision for education Gini. Cumulative cigarettes smoked over periods of both 15 and 20 years was not associated with the prevalence of vision severity.

Compared with the single-level analysis, two-level analysis (<50 years and ≥50 years) by age group provided a more nuanced statistical analysis across severities and near and distance impairment. In the two-level regression, associations become increasingly positive in the older age groups for second-hand smoke and women, which showed a stronger positive association than in older males. Education Gini inequality exhibited a strong association for all age-specific groups and less developed super-regions.

For smoking prevalence and blindness, the strong association for South East Asia across cohorts is discernible for all age groups and sexes. Super-regional findings are displayed by sex, age group and risk factor in Figs. S6 and S7 for blindness and MSVI, respectively.

Female (F): male (M) ratios were investigated for the prevalence of blindness and MSVI in 193 countries, comparing cross-temporally in 2020 vs 1990. There were significant positive slopes (~0.80) in the F:M prevalence of blindness and MSVI, indicating that the ratio has converged towards 1.0 globally and regionally over time, progressing closer to equal prevalence of blindness and MSVI in the two sexes (Figs. S8 and S9 of Appendix).

The rate of change in the prevalence of blindness and MSVI from 1990 to 2020 was also analysed in all persons, compared with the rate of change in the F:M ratio over the same period. A positive linear trend [coefficients 0.15–0.2] was observed, indicating that a reduction in overall rates of blindness and MSVI was associated with the reduction of sex inequity for these conditions (Figs. S8 and S9). However, this correlation did not reach statistical significance.

## Discussion

Prevalence and covariate data considered in the current analysis provide a powerful cross-sectional analysis for 2020. For the subset of plausible and well-studied risk factors for vision and their associations with VI prevalence and their relative change, it was attempted to strike a balance between analytical simplicity and spatio-temporal sophistication between individual and collective risk factors, rather than in the stronger sense of causal effect or contribution to a specific category of VI. Some of the main results appear to be borne out in recent epidemiological research, and their stability over the past three decades is encouraging. The analysis for the rates of change in 1990–2020 is also novel and offers additional insights compared with the cross-sectional analysis alone.

For contentious behavioural factors such as smoking and alcohol, the results show the same patterns for rates of change as for the cross-sectional analysis. Because of confounding, at the global level they are protective, and the association of rates of change is inverse; yet when considering regions with more prevalent smoking or alcohol consumption such as South East Asia, East Asia and Oceania and Latin America and the Caribbean, larger reductions in these risk factors from 1990–2020 are associated with greater declines in blindness prevalence, while increases in these risk factors are associated with lesser reductions. Given the constraints of considerable uncertainty and confounding when interpreting this relation as a dose-response [12], super-regional findings give a further novel perspective, so that they can be used to advocate for targeted policies.

Previous reports by VLEG in 2015 [3, 4] and VLEG/GBD in 2020 [1, 2] have observed blindness and MSVI to be more common globally in females than males, even after adjusting for the principal causes of cataract, diabetic retinopathy, age-related macular degeneration and undercorrected refractive error (except for glaucoma) [2]. The cross-sectional multiple variable analysis for 2020 demonstrated that female sex carried a higher risk of blindness and MSVI. A recent study of adults aged ≥ 50 years in six low- and middle-income countries using data from the Study on Global Ageing and Adult Health (2007–2010) [17] reported that distance VI was significantly associated with female sex [18], with 20–30% higher odds of having distance VI in China, Ghana, India and South Africa, but not in Mexico or Russia, comparable with the present findings with the exception of South Africa. Interestingly, women did not report greater receipt of eye care in countries where they were found to have excess VI. Improving gender equity is one of the UN Sustainable Development Goals (SDG 5) [19] and data on poorer access to eye health services among females allows for scaling of prioritised interventions such as community outreach vision screening services that have been shown to increase equity for females, marginalised communities and the elderly [20, 21].

At a cross-sectional level in 2020 for both sexes, there was a clear association between better education and less VI, which was consistent across super-regions and the three measures of education used here. South East Asia, East Asia and Oceania, Sub-Saharan Africa and Latin America and the Caribbean have stronger associations than High Income countries, especially for women and older age groups. The association of age cohort-specific educational inequity, as measured by the education Gini, further emphasised the importance of education. The analysis of change in education years per capita over three decades also showed that increasing education was associated with a decline in both blindness and various severities of VI. Many reports, usually within-country, have explored the association of education with vision loss, and interventional studies emphasise the success of correcting VI for academic test scores [22–24] and reading/word identification [25]. Access to education is another of the UN Sustainable Development Goals (SDG 4), and improving education remains critical to other development goals (SDGs 1, 2 and 8) [26].

In Figs. S1 and S2, it is shown that the super-regional associations tend to be consistent with the globally estimated associations, but there are regional exceptions as well as cultural and sex differences in risk factors. For smoking prevalence and VI, the single and multiple variable analyses showed a negative association (i.e., a protective effect) in adults aged 50+ years (for all vision loss severities except MSVI in men) at a global level which was most evident in women. This ‘protective’ effect of smoking prevalence also was found in the rates of change analysis between 1990 and 2020. Super-regionally, a strong positive association was observed between smoking and blindness prevalence for South East Asia and East Asia and Oceania, where the heavy consumption of tobacco products was a major risk factor among men (Fig. S2). This is reflected in a high prevalence of smoking and sex inequity in those regions, especially Indonesia, China and the Philippines. Similarly, while negative associations were globally found for alcohol consumption for bingers, nuanced super-regional analysis found a strong positive effect for binge drinking in Latin America and the Caribbean, where drinking is the single biggest driver of overall disease burden [27]. A recent genetic association study in France [13] found a 0.98 OR for moderate smokers (up to <20 cigarettes/day) with VI (mainly mild vision loss) and a 1.5 OR (corresponding to a +0.2 coefficient) for heavy smokers. It appears that in regions where the prevalence of smoking is high, as well as being high currently, the positive association becomes apparent at a population level with a similar relationship seen for heavy drinking, possibly linked via AMD and alcohol-induced Vitamin A Deficiency [28, 29].

Healthcare access measures such as HAQI and MCI were associated with reduced prevalence of blindness and VI in most super-regions (Fig. S1), and this was corroborated similarly when considering the percentage rate of change from 1990 to 2020 (Fig. S3). Regarding the prevalence of blindness, in the majority of the super-regions, with the exception of South Asia and Latin America and the Caribbean, the association was negative and particularly strong for South East Asia, East Asia and Oceania, which justifies the need for equitable investment in the training and local retention of healthcare professionals. Higher Vitamin A deficiency was associated with greater VI across populations, both globally and by super-region. Obesity was protective for vision loss, except for Sub-Saharan Africa, where a higher obesity prevalence was associated with greater VI prevalence.

Until recently, very little was published regarding the relationship between pollution and VI. A non-population-based study in the UK reported associations between ambient air pollution and self-reported AMD [30] and self-reported glaucoma [31]. Additionally, a nationwide cross-sectional study from China found higher odds of VI in children exposed to higher levels of air pollution [32]. This global analysis has demonstrated that indoor and outdoor pollution were positively associated with VI, with a stronger association at more severe levels of vision loss. Interestingly, with second-hand smoke coefficients, there were two age-specific regimes in the multilevel regression: generally a constant negative association up to 50 years of age, and then a positive association that became stronger with age, and also stronger for women than men. As with smoking and binge drinking, rates of change for outdoor pollution generally show a global protective effect, although a positive association was found in more heavily-polluted regions of South East Asia, East Asia and Oceania.

Finally, in terms of eye care services, this study explored the association between ophthalmologists per million population and the prevalence of VI at both global and super-regional levels. While at a global level, a negative association was found with blindness and MSVI; for most regions, there was a flat or even slightly positive association. Resnikoff et al. [14] reported that the density of ophthalmologists performing cataract surgery was very weakly correlated (*R*^2^ = 0.08) with the prevalence of blindness, with considerable inter-country variation. At a super-regional level, significant negative associations between ophthalmologists per million population and blindness and MSVI were found in the following regions: South East Asia, East Asia and Oceania; Central, Eastern Europe and Central Asia; Sub-Saharan Africa; North Africa and Middle East; Latin America. This may be of use in advocating for increased ophthalmologist coverage in these regions. A recent update to eye workforce figures and disaggregation of the workforce by profession [33] may provide more opportunity to explore such associations in the future.

There are limitations to this analysis. Modelling and age standardisation choices inherent in both VI estimates and covariates have a considerable impact on national results. In addition to the general limitations of the GBD as described recently elsewhere, there is the possibility of ‘ecological fallacy’ and the challenge of analysing and interpreting data at the aggregate versus individual level [34]. Reverse causation can also be a concern. Certainly, a recommendation arising from the current work would be to obtain individual-level risk factor data from the surveys included to compile a risk factor database which can be analysed, rather than drawing too much inference from macro risk factors which may or may not have relevance at an individual level for VI risk.

A major strength and contribution of this study is the confirmation of associations at a population level, both globally and super-regionally, for known epidemiologically-based risk factors related to vision loss where reliable estimates are available at sufficient granularity, and which appear to be stable over decades. In particular, the super-regional analysis of vision risk factors based on GBD 2019 data is novel.

Each country faces a unique set of environmental, social and economic circumstances that are risk factors for late presentation of eye disease, and these are by no means limited to those of low- and middle-income countries. Considerable research progress has been made in the last decade, with well-designed population-based studies of eye disease in more countries and an increased focus on risk factors for VI. Detailed knowledge of these factors within countries provides the opportunity to trial future interventions more effectively, not only by traditional eye care professionals but also with allied groups, such as educationalists, social care groups, specialists in diseases of the elderly (e.g., dementia and hearing loss) and economists, supported by patient and public participation.

## Supplementary Information

COMPLETE APPENDIX- revised

## Data Availability

Requests for access to data used for this analysis can be made by contacting the corresponding author.

## References

[1] GBD 2019 Blindness and Vision Impairment Collaborators, on behalf of the Vision Loss Expert Group of the Global Burden of Disease Study. Trends in prevalence of blindness and distance and near vision impairment over 30 years: an analysis for the Global Burden of Disease Study. Lancet Glob Health. 2021;e130–43. 10.1016/S2214-109X(20)30425-3.

[2] GBD 201 Blindness and Vision Impairment Collaborators, on behalf of the Vision Loss Expert Group of the Global Burden of Disease Study. Causes of blindness and vision impairment in 2020 and trends over 30 years and prevalence of avoidable blindness in relation to VISION 2020: the Right to Sight: an analysis for the Global Burden of Disease Study. Lancet Glob Health. 2021;9:e144–60. 10.1016/S2214-109X(20)30489-7.

[3] Bourne RRA, Flaxman SR, Braithwaite T, Cicinelli MV, Das A, Jonas JB, et al. Magnitude, temporal trends and projections of the global prevalence of blindness and distance and near vision impairment: a systematic review and meta-analysis. Lancet Glob Health. 2017;5:e888–97. 10.1016/S2214-109X(17)30293-0. 28779882

[4] Flaxman SR, Bourne RRA, Resnikoff S, Ackland P, Braithwaite T, Cicinelli MV, et al. Global causes of blindness and distance vision impairment 1990-2020: a systematic review and meta-analysis. Lancet Glob Health. 2017;5:e1221–34. 10.1016/S2214-109X(17)30393-5. 29032195

[5] Reed-Jones RJ, Solis GR, Lawson KA, Loya AM, Cude-Islas D, Berger CS. Vision and falls: a multidisciplinary review of the contributions of visual impairment to falls among older adults. Maturitas. 2013;75:22–8. 10.1016/j.maturitas.2013.01.019. 23434262

[6] Dhital A, Pey T, Stanford M. Visual loss and falls: a review. Eye. 2010;24:1437–46. 10.1038/eye.2010.60. 20448666

[7] Gupta P, Man REK, Fenwick EK, Qian C, Sim R, Majithia S, et al. Associations between visual impairment, incident falls and fall frequency among older Asians: longitudinal findings from the Singapore Epidemiology of Eye Diseases study. Br J Ophthalmol. 2022;107:1590–6. 10.1136/bjo-2021-320873. 35914927

[8] Livingston G, Huntley J, Liu KY, Costafreda SG, Selbæk G, Alladi S, et al. Dementia prevention, intervention and care: 2024 report of the *Lancet* standing Commission. Lancet. 2024;404:572–628. 10.1016/S0140-6736(24)01296-0. 39096926

[9] Lim ZW, Chee ML, Soh ZD, Cheung N, Dai W, Sahil T, et al. Association between visual impairment and decline in cognitive function in a multiethnic Asian population. JAMA Netw Open. 2020;3:e203560. 10.1001/jamanetworkopen.2020.3560. PMC7180417 32324240

[10] Vu TA, Fenwick EK, Gan ATL, Man REK, Tan BKJ, Gupta P, et al. The bidirectional relationship between vision and cognition: a systematic review and meta-analysis. Ophthalmology. 2021;128:981–92. 10.1016/j.ophtha.2020.12.010. PMC12962451 33333104

[11] Stevens GA, Bennett JE, Hennocq Q, Lu Y, De-Regli LM, Rogers L, et al. Trends and mortality effects of vitamin A deficiency in children in 138 low-income and middle-income countries between 1991 and 2013: a pooled analysis of population-based surveys. Lancet Glob Health. 2015;3:e528–36. 10.1016/S2214-109X(15)00039-X. 26275329

[12] Fan AZ, Li Y, Zhang X, Klein R, Mokdad AH, Saaddine JB, et al. Alcohol consumption, drinking pattern and self-reported visual impairment. Ophthalmic Epidemiol. 2012;19:8–15. 10.3109/09286586.2011.591037. 22273354

[13] Merle BMJ, Moreau G, Ozguler A, Srour B, Cougnard-Grégoire A, Goldberg M, et al. Unhealthy behaviours and risk of visual impairment: The CONSTANCES population-based cohort. Sci Rep. 2018;8:6569. 10.1038/s41598-018-24822-0. PMC5920045 29700371

[14] Resnikoff S, Lansingh VC, Washburn L, Felch W, Gauthier T-M, Taylor HR, et al. Estimated number of ophthalmologists worldwide (International Council of Ophthalmology update): will we meet the needs. Br J Ophthalmol. 2020;104:588–92. 10.1136/bjophthalmol-2019-314336. PMC7147181 31266774

[15] International Classification of Diseases 11th Revision. [cited 2026 Jan 2]. Available from: https://icd.who.int/en.

[16] The World Bank. Gini Index. [cited 2026 Jan 2]. Available from: https://data.worldbank.org/indicator/SI.POV.GINI.

[17] World Health Organization. WHO Study on global AGEing and adult health (SAGE). [cited 2026 Jan 2]. Available from: https://www.who.int/data/data-collection-tools/study-on-global-ageing-and-adult-health.

[18] Ehrlich JR, Stagg BC, Andrews C, Kumagai A, Musch DC. Vision impairment and receipt of eye care among older adults in low- and middle-income countries. JAMA Ophthalmol. 2019;1372:146–58. 10.1001/jamaophthalmol.2018.5449. PMC6440432 30477016

[19] United Nations. Department of Economic and Social Affairs. Sustainable Development. [cited 2026 Jan 2]. Available from: https://sdgs.un.org.

[20] Zhang M, Wu J, Li L, Xu D, Lam DSC, Lee J, et al. Impact of cataract screening outreach in rural China. Invest Ophthalmol Vis Sci. 2010;51:110–14. 10.1167/iovs.09-4190. 19797234

[21] Liang Y, Jiang J, Ou W, Peng X, Sun R, Xu X, et al. Effect of community screening on the demographic makeup and clinical severity of glaucoma patients receiving care in urban China. Am J Ophthalmol. 2018;195:1–7. 10.1016/j.ajo.2018.07.013. 30053479

[22] Glewwe P, West KL, Lee J. The impact of providing vision screening and free eyeglasses on academic outcomes: evidence from a randomized trial in title I elementary schools in Florida. J Policy Anal Manag. 2018;37:265–300. 10.1002/pam.22043. PMC5959017 29693366

[23] Hannum E, Zhang Y. Poverty and proximate barriers to learning: vision deficiencies, vision correction and educational outcomes in rural northwest China. World Dev. 2012;40:1921–31. 10.1016/j.worlddev.2012.04.029. PMC4655323 26609193

[24] Hark LA, Thau A, Nutaitis A, Mayro EL, Zhan T, Dabbish N, et al. Impact of eyeglasses on academic performance in primary school children. Can J Ophthalmol. 2020;55:52–57. 10.1016/j.jcjo.2019.07.011. 31712026

[25] Fireison CK, Moore JE. Employment outcomes and educational backgrounds of legally blind adults employed in sheltered industrial settings. J Vis Impair Blind. 1998;92:740–47. 10.1177/0145482X9809201102.

[26] Cremin P, Nakabugo MG. Education, development and poverty reduction: a literature critique. Int J Educ Dev. 2012;32:499–506. 10.1016/j.ijedudev.2012.02.015.

[27] Rehm J, Monteiro M. Alcohol consumption and burden of disease in the Americas: implications for alcohol policy. Rev Panam Salud Publica. 2005;18:241–8. 10.1590/s1020-49892005000900003. 16354420

[28] Kuan V, Warwick A, Hingorani A, Tufail A, Cipriani V, Burgess S, et al. Association of smoking, alcohol consumption, blood pressure, body mass index and glycemic risk factors with age-related macular degeneration: a Mendelian randomization study. JAMA Ophthalmol. 2021;139:1299–306. 10.1001/jamaophthalmol.2021.4601. PMC8569599 34734970

[29] Clugston RD, Blaner WS. The adverse effects of alcohol on vitamin A metabolism. Nutrients. 2012;4:356–71. 10.3390/nu4050356. PMC3367262 22690322

[30] Chua SYL, Warwick A, Peto T, Balaskas K, Moore AT, Reisman C, et al. Association of ambient air pollution with age-related macular degeneration and retinal thickness in UK Biobank. Br J Ophthalmol. 2022;106:705–11. 10.1136/bjophthalmol-2020-316218. 33495162

[31] Chua SYL, Khawaja AP, Morgan J, Strouthidis N, Reisman C, Dick AD, et al. The relationship between ambient atmospheric fine particulate matter (PM_2.5_) and glaucoma in a large community cohort. Invest Ophthalmol Vis Sci. 2019;60:4915–23. 10.1167/iovs.19-28346. 31764948

[32] Yang B, Guo Y, Zou Z, Gui Z, Bao W, Hu L, et al. Exposure to ambient air pollution and visual impairment in children: a nationwide cross-sectional study in China. J Hazard Mater. 2021;407:124750. 10.1016/j.jhazmat.2020.124750. 33341569

[33] Resnikoff S, Block SS, Chai S, Gammoh Y, Hendicott P, Stern J, et al. The global eye care workforce: 2023 estimates across ophthalmologists, optometrists and allied personnel. AJO Int. 2026;3:100260. 10.1016/j.ajoint.2026.100260.

[34] Subramanian SV, Jones K, Kaddour A, Krieger N. Revisiting Robinson: the perils of individualistic and ecologic fallacy. Int J Epidemiol. 2009;38:342–60. 10.1093/ije/dyn359. PMC2663721 19179348

