# Supplementary material for: Global and Regional Risk Factors for Vision Impairment in 2020 and Multi-decadal Trends 1990–2020: A Meta-Analysis and Visual Display Based on Prevalence Data From the Global Burden of Disease study and Vision Loss Expert Group
Source: Ophthalmic Physiol Opt. 2026 Oct 16;46(6):1262–71. doi: 10.1007/s44402-026-00208-y (PMC13694339; doi:10.1007/s44402-026-00208-y)
Supplement: Supplementary file 1 [file 44402_2026_208_MOESM1_ESM.pdf]

Appendix Text S1

We preliminarily screened a database of covariates used across GBD modelling for those covariates with a potential link to vision-related disease:

https://ghdx.healthdata.org/record/ihme-data/gbd-2023-covariates-1980-2023

Our research team selected 24 covariates a priori that were stratified by age and sex after considering the available literature (a data dictionary is presented below in Table 1). Overall, the population risk factors for vision considered in our work included a mixture of behavioural, physiological, socio-demographic, and environmental groups of covariates, ranging from common behavioural risks such as smoking (measured as smoking prevalence, second-hand smoking prevalence, per capita and cumulative cigarette consumption), alcohol consumption (in terms of daily intake, binge drinking and pregnancy alcohol consumption, and current drinking proportion), nutrition (vitamin A intake and deficiency), physiological markers (adult body mass index [BMI], diabetes prevalence), proxies for educational attainment (education years per capita and proportion of population with zero years of education), quality and access indices for healthcare, such as HAQI (health access and quality index) and maternal care and immunisation, environmental factors such as pollution (both outdoor and indoor) and proportion of access to sanitation, and socio-demographic and inequality indices, such as socio-demographic index (SDI) and education Gini (a measure of education inequality), which were supplemented with country estimates for the number of ophthalmologists per million population and Gini index, referred hereon as income GINI. Almost all these estimates, with the exception of the latter, came from repeated monitoring and modelling surveys that have been held at least annually and provided an opportunity to assign a portion of the vision impairment and disease burden trends to the corresponding risk factor trends in the whole population.

Table 1. Covariates used in the risk factor analysis, presented as a Data Dictionary

Name of covariate	Description (including units)
**Where data is available only for both sexes**	
**Smoking Prevalence (Both)**	Smoking prevalence (proportion between 0 and 1) - age standardised for both sexes
**Outdoor Pollution**	Population weighted average of PM2.5 (micrograms / m^3), between 0 and 100
**Maternal Care and Immunisation**	A measure of health system access estimated using a principal component analysis of antenatal clinics, DTP3 immunization, measles immunization, in-facility delivery, and skilled birth attendance. Maximum value for each year capped to min OECD value.
**Healthcare Access & Quality Index**	Healthcare Access & Quality Index (between 0 and 100)
**Cigarettes Per Capita**	Number of cigarettes or cigarette equivalents consumed per adult aged 15 years or older per year
**Vit.A Units Unadjusted**	Total Vitamin A Retinol Activity Equivalents ug/p/day unadjusted
**Sanitation Proportion**	Percent of population with access to improved toilet types as defined by the Joint Monitoring Program
**Socio Demographic Index**	A measure of development estimated via principal component analysis using log-transformed LDI, TFR, and education years per capita over age 15
**Indoor Pollution**	Household prevalence of cooking with coal or biomass
**Ophthalmologists P.M.P.**	Number of Opthalmologists, per Million population, from 2015 estimates by Serge Resnikoff et. al
**Where data is available for men and women separately**	
**Smoking Prevalence**	Smoking prevalence (proportion between 0 and 1) - age standardised for each sex
**Alcohol Binge Drinker Proportion**	Alcohol binge drinker proportion [0-1], age-standardized
**Alcohol Grams per Day**	Mean age-standardized alcohol consumption, measure in grams per day
**Diabetes Prevalence**	Age-standardized prevalence of diabetes
**Zero Education Proportion**	Proportion of the population with 0 years of education, age-standardized
**Education Years per Capita**	Age standardized level of educational attainment, in years
**Maternal Alcohol Consumption**	Country-level covariate for Foetal Alcohol Syndrome. All subnational estimates imputed with the country-level estimates. Non-sovereign locations, TWN, PSE, AND, SYR, and PRK did not have data and were assigned estimates for similar countries.
**Mean Adult BMI**	Mean body mass index (kg/m^2) for males and females above age 20
**Obesity Prevalence**	Prevalence of obesity (age-standardized)
**Vitamin A Deficiency Prevalence**	Age-standardized prevalence of vitamin A deficiency
**Where data is available for men and**	

**women, and also by 5 year age groups (0-99)**	
**Smoking Prevalence (Age-specific)**	Smoking prevalence (proportion between 0 and 1) - age specific for males and females
**Secondhand Smoke**	Proportion of population exposed to secondhand smoke, derived from risk factor estimate
**Current Drinkers Prevalence**	Prevalence of current drinkers from (GBD 2016) Alcohol Use Estimates 1990-2016.
**Education Years Per Capita**	Mean level of educational attainment by 5 year age cohort
**Cumulative Cigarettes (15Y)**	Total number of cigarettes or cigarette equivalents smoked by an age cohort in the past 15 years (Estimates assumed to be zero for age-groups 2-6)
**Cumulative Cigarettes (20Y)**	Total number of cigarettes or cigarette equivalents smoked by an age cohort in the past 20 years (Estimates assumed to be zero for age-groups 2-6)
**Education Gini Index**	Education Relative Inequality (Gini)
**Covariates Rates of Change**	These are defined as relative percentage change from 1990 to 2020 (and 1990-2000 and 1990-2010 for the sensitivity analysis) for the sets of covariates above. Unless otherwise specified, nonnormalised units.

Appendix Text S2

- more detailed than in manuscript section

Definitions and Statistical Methods

The Institute for Health Metrics and Evaluation (IHME) and the VLEG produced updated prevalence estimates for vision impairment for several countries, regions and macroregions, recorded at five-year time intervals and using the combined MR-BRT and DisMod models as described recently.[1] While we note that many of the data to which this study refers to come from aggregations and are at best modelled estimates, we developed a methodology to investigate both similarities and substantial discrepancies in temporal trends across countries and their social and health determinants and performed statistical analyses to assess the contribution of several risk factors for VI and their relative changes over the three intervening decades. To make the analysis comparable and to account for the demographic risk factors of age and sex, we used all ages and 50+ age-standardised mean estimates for both prevalence and covariates where possible. We further stratified covariates into three criteria: factors where covariates referred to all persons; factors pertaining to each sex disaggregated, and those factors available at the granularity of five-year age groups between 1-4 and 95+ for males and females.

We used International Classification of Diseases 11th edition criteria for vision loss, as used by WHO, which categorises people according to vision in the better eye on presentation, in which mild VI is defined as a visual acuity of 6/18 or better but less than 6/12, moderate VI as a visual acuity of 6/60 or better but less than 6/18, severe VI as a visual acuity of 3/60 or better but less than 6/60 (hence our definition of moderate and severe VI is <6/18 to 3/60), and blindness as a visual acuity of less than 3/60 or less than 10° visual field around central fixation (although the visual field definition is rarely used in population-based eye surveys). VI from uncorrected presbyopia was defined as presenting near vision of worse than N6 or N8 at 40 cm when best-corrected distance visual acuity was 6/12 or better.

We analysed the association of risk factors with crude and age-standardised estimates of blindness and VI in terms of all-cause prevalence in 2020 separately for each severity as follows: blindness (BI), moderate and severe VI (MSVI), mild VI (MVI), and near vision impairment due to uncorrected presbyopia (NVI). Additionally, the trend contribution of the percentage rates of change in risk factors to the rates of change in prevalence between 1990-2020 were analysed.

Wherever possible, for the non-specific age groups and risk factors, age-standardised covariate estimates were used, with central estimates taken as observation points at the country level, for a total of 193 countries in the VLEG-GBD vision database (all sovereign states in the ISO-alpha3 coding system). This introduces a source of randomness into the country measurement, yet the use of mean estimates in population-based country analyses is well established.

For each risk factor and group of covariates, we estimated the global mean associations and their 95% uncertainty intervals (UI) using posterior draws under a generalised linear model, examining both unweighted regressions and coefficient values weighted by country population. In addition to global estimates, we also calculated associations for the seven GBD super-regions of High Income (Australasia, Western Europe, Asia-Pacific & North America); Latin America & Caribbean; Sub-Saharan Africa; South Asia; South East Asia, East Asia & Oceania; North Africa & Middle East; and Central Europe, Eastern Europe & Central Asia.

For all associations computed, the dependent variable was either one of the four VI severity prevalences in 2020 using GBD2019 estimates, and its relative percentage rate of change between 1990 and 2020, and the covariate (or its rate of change from 1990, respectively) measured in that year or, where missing, using the last available estimate for the preceding years. Three models were fitted, specified as a generalised linear model of a) covariates available for all persons, b) women and men separately, and for the age-specific estimates, c) as a model specified at the single level of men and women and at the further level of 5-year age cohort. The multivariable analysis

considered the joint effect of all factors in the model, compared to a simple univariable analysis of a single factor.

To disentangle the effects of uncertainty in mean estimates and temporal variations and to make our analysis more robust when calculating the contribution of the risk factor trends to impairment, we also assessed global and regional risk factors at intermediate decadal checkpoints at years 2000 and 2010 for each vision impairment prevalence and risk factors estimate as well as 1990 and 2020. We also performed estimates for the associations of the rates of relative change for the 1990-2000 and 1990-2010 time periods in addition to the baseline 1990-2020 change. Finally, we made further sensitivity adjustments by weighting each regression by country population.

To obtain odds ratios (OR) for Blindness, MSVI, Mild and Near Vision impairments respectively, we calculated the results for significant and non-significant single and multiple variable risk factors for 50+ age-standardised prevalence in 2020 via the exponentiated coefficients of a series of generalised linear regressions on each of four VI outcomes for covariate estimates that were available for all persons, and for women and men separately. Further single-level and nested regressions were performed on age-specific estimates, of which the pooled coefficients for education Gini index and second-hand smoke prevalence are also produced. The outputs of the regression coefficients are interpreted as follows: for each change in either units or proportions of the covariate, under the assumptions of the statistical model one can expect a given – positive or negative – percentage change in prevalence. This allows a direct interpretation in terms of odds ratios for their effect: a 0.98 OR, for example, corresponds to a 2% reduction in risk compared to no impairment (a 0.02 coefficient in the regression)

All statistical analyses were performed in R version 3.6.3 using the *stan_glm* function (*stan_glmer* for the two-level age-specific hierarchical model) in the *rstanarm* package with the default of Gaussian priors on the intercept and coefficient and exponential distribution for sigma. Figures were produced using the *ggplot2* R package.

Reference:

1. GBD 2019 Blindness and Vision Impairment Collaborators, on behalf of the Vision Loss Expert Group of the Global Burden of Disease Study. Trends in prevalence of blindness and distance and near vision impairment over 30 years: an analysis for the Global Burden of Disease Study. Lancet Global Health 2021. Vol 9(2) e130-e143.

Appendix Tables S1-S3

Table S1. Global Risk Factors associated with age-standardised prevalence of blindness (BI), moderate and severe (MSVI), Mild and Near Vision impairments for 193 countries: Single and multiple variable odds ratios and 95% UI for **All Persons.**

Univariable analysis: 50 PLUS AGE-STANDARDISED ALL CAUSES, 2020												
**Prevalence 2020**	**Model: Blindness**			**Model: MSVI**			**Model: Mild**			**Model: Near Vision**		
**Covariate**	**OR**	**2.50%**	**97.50%**	**OR**	**2.50%**	**97.50%**	**OR**	**2.50%**	**97.50%**	**OR**	**2.50%**	**97.50%**
Smoking Prevalence (Both)	0.9235	0.9028	0.9452	0.9224	0.8584	0.9906	0.8588	0.8223	0.8984	0.6925	0.5583	0.8621
Outdoor Pollution	1.0004	1.0003	1.0005	1.0012	1.0009	1.0014	1.0004	1.0002	1.0006	1.0024	1.0015	1.0033
Maternal Care and Immunisation	0.9916	0.9904	0.9928	0.9837	0.9794	0.9878	0.9874	0.9847	0.9903	0.9448	0.9326	0.9571
Healthcare Access & Quality Index	0.9995	0.9995	0.9996	0.999	0.9988	0.9992	0.9991	0.999	0.9992	0.9959	0.9954	0.9965
Cigarettes Per Capita	1	1	1	1	1	1	1	1	1	0.9999	0.9999	0.9999
Vit.A Units Unadjusted	1	1	1	1	1	1	1	1	1	1	1	1
Sanitation Proportion	0.9609	0.9544	0.9672	0.9438	0.9224	0.9666	0.9416	0.9277	0.9557	0.7153	0.6731	0.7592
Socio Demographic Index	0.9429	0.9347	0.9511	0.8852	0.8602	0.911	0.8927	0.878	0.9076	0.6137	0.5684	0.6624
Indoor Pollution	1.026	1.0213	1.0306	1.0474	1.0312	1.0633	1.0461	1.0358	1.056	1.2593	1.2083	1.3109
Ophthalmologists P.M.P.	0.9998	0.9997	0.9998	0.9995	0.9994	0.9996	0.9995	0.9994	0.9996	0.9984	0.998	0.9988
**Rates of Change 1990-2020**	**Model: Blindness**			**Model: MSVI**			**Model: Mild**			**Model: Near Vision**		
**Covariate**	**OR**	**2.50%**	**97.50%**	**OR**	**2.50%**	**97.50%**	**OR**	**2.50%**	**97.50%**	**OR**	**2.50%**	**97.50%**
Smoking Prevalence (Both) Rate of Change	0.9212	0.8558	0.9944	0.9803	0.9547	1.0067	0.9668	0.9413	0.9923	0.9679	0.9334	1.0031
Outdoor Pollution Rate of Change	0.7669	0.6891	0.8543	0.9257	0.8892	0.9619	0.9247	0.8895	0.9636	0.8573	0.8163	0.9002
Maternal Care and Immunisation Rate of Change	0.9999	0.9999	1	1	1	1	1	1	1	1	1	1
Healthcare Access & Quality Index Rate of Chang	0.8894	0.8642	0.9161	0.9806	0.9697	0.9916	0.9576	0.9472	0.9675	0.9605	0.9466	0.9745
Cigarettes Per Capita Rate of Change	0.9387	0.8599	1.0204	0.983	0.9544	1.0125	0.9709	0.9433	1.001	0.9842	0.9455	1.0242
Vit.A Units Unadjusted Rate of Change	0.975	0.959	0.9911	0.9947	0.9884	1.0006	0.9913	0.9851	0.9974	0.9886	0.9808	0.9967
Sanitation Proportion Rate of Change	0.9891	0.9805	0.9975	0.9963	0.9935	0.9992	0.994	0.9912	0.9969	0.9949	0.991	0.9988
Socio Demographic Index Rate of Change	0.8869	0.8557	0.9192	0.9725	0.9591	0.986	0.9496	0.9381	0.9617	0.9376	0.9217	0.9538
Indoor Pollution Rate of Change	1.0729	1.018	1.1288	1.0015	0.9838	1.02	0.9898	0.9716	1.0074	1.0231	0.9988	1.0484
**Multivariable analysis: 50PLUS AGE-STANDARDISED ALL CAUSES, 2020**												
**Prevalence 2020**	**Model: Blindness**			**Model: MSVI**			**Model: Mild**			**Model: Near Vision**		
**Covariate**	**OR**	**2.50%**	**97.50%**	**OR**	**2.50%**	**97.50%**	**OR**	**2.50%**	**97.50%**	**OR**	**2.50%**	**97.50%**
Smoking Prevalence (Both)	0.9915	0.966	1.0166	1.1509	1.0617	1.2494	1.014	0.9678	1.0613	1.1521	0.9095	1.4632
Outdoor Pollution	1.0002	1.0001	1.0003	1.0009	1.0006	1.0012	1	0.9998	1.0001	1.0007	0.9999	1.0015
Maternal Care and Immunisation	0.9977	0.9956	0.9998	1	0.9929	1.0072	1.0047	1.0005	1.0087	1.0361	1.0156	1.0583
Healthcare Access & Quality Index	0.9998	0.9997	1	0.9993	0.9988	0.9999	0.9995	0.9992	0.9998	0.9949	0.9932	0.9964
Cigarettes Per Capita	1	1	1	1	1	1	1	1	1	1	1	1.0001
Vit.A Units Unadjusted	1	1	1	1	1	1	1	1	1	1	1	1
Sanitation Proportion	0.9928	0.9817	1.0038	1.054	1.0156	1.0951	1.0273	1.0061	1.0487	0.8836	0.7886	0.9857
Socio Demographic Index	0.9963	0.9746	1.019	0.938	0.8701	1.0113	0.9369	0.8982	0.9778	1.0274	0.8373	1.2729
Indoor Pollution	0.9989	0.9903	1.0073	0.9912	0.9652	1.0191	0.9978	0.981	1.014	1.0286	0.9469	1.1148
Ophthalmologists P.M.P.	1	0.9999	1	0.9998	0.9997	1	0.9999	0.9998	1	1.0002	0.9997	1.0007
**Rates of Change 1990-2020**	**Model: Blindness**			**Model: MSVI**			**Model: Mild**			**Model: Near Vision**		
**Covariate**	**OR**	**2.50%**	**97.50%**	**OR**	**2.50%**	**97.50%**	**OR**	**2.50%**	**97.50%**	**OR**	**2.50%**	**97.50%**
Smoking Prevalence (Both) Rate of Change	0.9414	0.8533	1.0404	0.9894	0.9472	1.0346	0.9818	0.9425	1.0226	0.9657	0.92	1.0153
Outdoor Pollution Rate of Change	0.8726	0.7841	0.97	0.95	0.9064	0.9941	0.9932	0.9513	1.0371	0.923	0.8776	0.9726
Maternal Care and Immunisation Rate of Change	1	0.9999	1	1	1	1	1	1	1	1	1	1
Healthcare Access & Quality Index Rate of Chang	0.9021	0.8664	0.9406	0.9915	0.9741	1.0086	0.9708	0.9552	0.9865	0.9979	0.978	1.0191
Cigarettes Per Capita Rate of Change	1.0115	0.907	1.1263	1.0052	0.9587	1.0555	1.0059	0.9625	1.051	1.0383	0.9824	1.0945
Vit.A Units Unadjusted Rate of Change	0.9938	0.9797	1.0082	0.9986	0.9925	1.0047	0.9992	0.9936	1.0048	0.999	0.9922	1.006
Sanitation Proportion Rate of Change	0.9907	0.9833	0.9983	0.9973	0.9939	1.0008	0.9972	0.9942	1.0002	0.997	0.9933	1.0007
Socio Demographic Index Rate of Change	0.9885	0.9301	1.0511	0.9887	0.9632	1.0147	0.9771	0.9536	1.0006	0.9404	0.9123	0.9678
Indoor Pollution Rate of Change	1.1896	1.1333	1.2465	1.0222	1.001	1.0437	1.0236	1.0052	1.0431	1.0717	1.0481	1.0968

Table S2. Global Risk Factors associated with age-standardised prevalence of blindness (BI), moderate and severe (MSVI), Mild and Near Vision impairments for 193 countries: Single and multiple variable odds ratios and 95% UI for **Females and Males.**

	**Univariable analysis: 50 PLUS AGE STANDARDISED ALL CAUSES, 2020**												
	**Prevalence 2020**	**Model: Blindness**			**Model: MSVI**			**Model: Mild**			**Model: Near Vision**		
**Sex**	**Covariate**	**OR**	**2.50%**	**97.50%**	**OR**	**2.50%**	**97.50%**	**OR**	**2.50%**	**97.50%**	**OR**	**2.50%**	**97.50%**
**MALE**	Smoking Prevalence	0.9719	0.9558	0.9894	1.0438	0.9922	1.0995	0.9689	0.9379	1.0009	0.9419	0.806	1.1022
**FEMALE**	Smoking Prevalence	0.8999	0.8809	0.9197	0.7895	0.7442	0.8382	0.794	0.765	0.8251	0.5356	0.4388	0.6573
**MALE**	Alcohol Binge Drinker Proportion	0.9599	0.9528	0.9677	0.8797	0.8597	0.9004	0.9267	0.9124	0.9411	0.8262	0.7584	0.9005
**FEMALE**	Alcohol Binge Drinker Proportion	0.9504	0.9386	0.9621	0.8548	0.8273	0.8829	0.8885	0.8698	0.9083	0.7508	0.6719	0.8369
**MALE**	Alcohol Grams per Day	0.9997	0.9994	0.9999	0.9986	0.998	0.9992	1	0.9996	1.0004	0.9993	0.9975	1.0012
**FEMALE**	Alcohol Grams per Day	0.9997	0.9993	1	0.9985	0.9976	0.9993	1.0004	0.9998	1.0011	0.9991	0.9962	1.0019
**MALE**	Diabetes Prevalence	1.0026	0.9532	1.0542	1.3594	1.1823	1.5654	1.1757	1.0678	1.3031	0.9575	0.6079	1.4984
**FEMALE**	Diabetes Prevalence	1.0411	0.9826	1.1034	1.4584	1.2504	1.6913	1.2646	1.1466	1.3954	0.9853	0.5985	1.5868
**MALE**	Zero Education Proportion	1.0641	1.0544	1.0739	1.129	1.0922	1.1694	1.09	1.0671	1.1137	1.3813	1.2475	1.5346
**FEMALE**	Zero Education Proportion	1.0547	1.047	1.0621	1.1034	1.0764	1.1295	1.0755	1.0581	1.0939	1.3022	1.2006	1.4126
**MALE**	Education Years per Capita	0.9965	0.996	0.997	0.9918	0.9902	0.9935	0.9932	0.9923	0.994	0.976	0.9709	0.9811
**FEMALE**	Education Years per Capita	0.9965	0.9961	0.997	0.9932	0.9917	0.9947	0.9937	0.9929	0.9946	0.9788	0.9743	0.9834
**MALE**	Maternal Alcohol Consumption	0.9367	0.9223	0.9518	0.8196	0.7818	0.859	0.8791	0.8539	0.9051	0.8213	0.699	0.9624
**FEMALE**	Maternal Alcohol Consumption	0.9264	0.9088	0.9435	0.8186	0.776	0.8635	0.8649	0.8331	0.8976	0.8081	0.6747	0.961
**MALE**	Mean Adult BMI	0.9967	0.996	0.9974	0.9935	0.991	0.996	0.9933	0.9919	0.9947	0.9701	0.9638	0.9762
**FEMALE**	Mean Adult BMI	0.9972	0.9962	0.9982	0.9969	0.994	0.9997	0.9941	0.9923	0.996	0.9805	0.9721	0.9892
**MALE**	Obesity Prevalence	0.9301	0.9096	0.952	0.8685	0.8102	0.9298	0.8392	0.8052	0.8731	0.4507	0.3761	0.5397
**FEMALE**	Obesity Prevalence	0.9515	0.9276	0.9748	0.9639	0.8945	1.037	0.8804	0.8399	0.924	0.6409	0.516	0.7952
**MALE**	Vitamin A Deficiency Prevalence	1.1274	1.104	1.1525	1.2469	1.1553	1.3433	1.1955	1.141	1.2507	2.7375	2.2571	3.3441
**FEMALE**	Vitamin A Deficiency Prevalence	1.1395	1.1077	1.1715	1.2201	1.1187	1.3314	1.2064	1.1401	1.2757	3.3669	2.7063	4.1896
	**Rates of Change 1990-2020**	**Model: Blindness**			**Model: MSVI**			**Model: Mild**			**Model: Near Vision**		
**Sex**	**Covariate**	**OR**	**2.50%**	**97.50%**	**OR**	**2.50%**	**97.50%**	**OR**	**2.50%**	**97.50%**	**OR**	**2.50%**	**97.50%**
**MALE**	Smoking Prevalence Rate of Change	0.9094	0.8465	0.9809	0.9771	0.9508	1.0046	0.9626	0.9379	0.9877	0.9573	0.9229	0.9934
**FEMALE**	Smoking Prevalence Rate of Change	0.9698	0.9017	1.0426	0.9889	0.9639	1.0151	0.9766	0.9515	1.0026	0.9898	0.9576	1.023
**MALE**	Alcohol Binge Drinker Proportion Rate of	1.1841	1.0012	1.3941	1.0255	0.9674	1.0913	1.0822	1.022	1.1483	1.0619	0.978	1.1508
**FEMALE**	Alcohol Binge Drinker Proportion Rate of	1.0371	0.9454	1.14	1.0331	1.0017	1.0649	1.0705	1.0364	1.1051	1.0315	0.9881	1.0767
**MALE**	Alcohol Grams per Day Rate of Change	1.7078	1.1811	2.481	1.0746	0.9349	1.2334	1.0279	0.8983	1.1787	0.9907	0.8241	1.1939
**FEMALE**	Alcohol Grams per Day Rate of Change	0.9601	0.669	1.3811	0.9448	0.8293	1.0745	0.9804	0.8541	1.1262	0.9295	0.7827	1.0976
**MALE**	Diabetes Prevalence Rate of Change	0.9548	0.8786	1.0363	0.961	0.9338	0.9897	0.9583	0.9301	0.987	0.9582	0.9204	0.9969
**FEMALE**	Diabetes Prevalence Rate of Change	0.9508	0.8779	1.0314	0.9638	0.9375	0.9904	0.9674	0.9397	0.9953	0.9555	0.9214	0.9918
**MALE**	Zero Education Proportion Rate of Chang	1.0495	0.9969	1.1046	1.005	0.9865	1.0243	1.0197	1.0009	1.0395	1.0117	0.9847	1.0383
**FEMALE**	Zero Education Proportion Rate of Chang	1.0483	0.981	1.1223	0.9887	0.9662	1.0119	1.0164	0.9918	1.0421	0.9859	0.9556	1.0185
**MALE**	Education Years per Capita Rate of Change	0.9293	0.9007	0.9589	0.9782	0.9676	0.9892	0.9707	0.9598	0.9814	0.9602	0.9462	0.9744
**FEMALE**	Education Years per Capita Rate of Change	0.9688	0.9549	0.9823	0.987	0.982	0.992	0.9882	0.9832	0.9931	0.9794	0.9732	0.9857
**MALE**	Maternal Alcohol Consumption Rate of Ch	1.0242	0.1538	6.6187	0.9532	0.4865	1.8394	0.7611	0.4069	1.4693	0.8507	0.3389	2.0858
**FEMALE**	Maternal Alcohol Consumption Rate of Ch	1.0482	0.1571	7.6015	1.0231	0.5113	2.0846	0.7083	0.3382	1.4661	0.7811	0.3138	1.955
**MALE**	Mean Adult BMI Rate of Change	0.263	0.1615	0.4311	0.9172	0.7604	1.0982	0.7874	0.6595	0.9455	0.5678	0.4508	0.7194
**FEMALE**	Mean Adult BMI Rate of Change	0.3615	0.241	0.5533	0.8838	0.7582	1.0291	0.6537	0.5665	0.7566	0.5957	0.4947	0.7196
**MALE**	Obesity Prevalence Rate of Change	0.9807	0.975	0.9864	0.9981	0.9959	1.0004	0.9934	0.9912	0.9956	0.9927	0.9898	0.9957
**FEMALE**	Obesity Prevalence Rate of Change	0.9783	0.9701	0.9866	0.9974	0.9943	1.0005	0.99	0.9871	0.9929	0.9898	0.9861	0.9937
**MALE**	Vitamin A Deficiency Prevalence Rate of Ch	1.4368	1.3208	1.5597	1.0518	1.0166	1.0888	1.1101	1.0758	1.1457	1.1606	1.1112	1.2122
**FEMALE**	Vitamin A Deficiency Prevalence Rate of Ch	1.3728	1.2589	1.501	1.0421	1.007	1.0793	1.1162	1.0803	1.1543	1.1663	1.1192	1.2128
	**Multivariable analysis: 50 PLUS AGE STANDARDISED ALL CAUSES, 2020**												
	**Prevalence 2020**	**Model: Blindness**			**Model: MSVI**			**Model: Mild**			**Model: Near Vision**		
**Sex**	**Covariate**	**OR**	**2.50%**	**97.50%**	**OR**	**2.50%**	**97.50%**	**OR**	**2.50%**	**97.50%**	**OR**	**2.50%**	**97.50%**
**MALE**	Smoking Prevalence	0.9942	0.9819	1.0061	1.0604	1.0196	1.1013	0.9804	0.9594	1.0018	1.0836	0.9468	1.2385
	Alcohol Binge Drinker Proportion	0.9913	0.9815	1.0013	0.955	0.9236	0.9867	0.9781	0.9613	0.9953	0.9664	0.8637	1.0783
	Alcohol Grams per Day	0.9999	0.9997	1	0.9986	0.9982	0.9991	1.0002	1	1.0005	0.9995	0.9977	1.0012
	Diabetes Prevalence	0.9937	0.9533	1.0372	1.4074	1.2302	1.6139	1.0635	0.9877	1.146	1.0288	0.6448	1.6619
	Zero Education Proportion	1.0162	0.9999	1.0331	0.9857	0.9349	1.0381	0.9285	0.9021	0.9559	0.7772	0.6488	0.9267
	Education Years per Capita	0.9988	0.9978	0.9998	0.9952	0.9919	0.9986	0.9923	0.9905	0.9941	0.9784	0.9674	0.9897
	Maternal Alcohol Consumption	0.9849	0.9693	1.0011	0.9942	0.9408	1.0484	0.9648	0.9383	0.9935	1.1488	0.9553	1.3814
	Mean Adult BMI	0.998	0.9963	0.9997	0.9947	0.9894	1.0002	0.9969	0.994	1	0.9851	0.9666	1.0042
	Obesity Prevalence	1.059	1.0124	1.1079	1.0894	0.9416	1.2564	1.0086	0.9302	1.0931	1.1147	0.6908	1.8492
	Vitamin A Deficiency Prevalence	1.0497	1.0245	1.0759	1.0044	0.9255	1.0893	0.973	0.9309	1.0158	1.692	1.2826	2.2634
**FEMALE**	Smoking Prevalence	0.9719	0.9506	0.9943	0.9148	0.8537	0.979	0.8851	0.8516	0.9181	0.7194	0.5733	0.9065
	Alcohol Binge Drinker Proportion	0.9916	0.9768	1.0063	0.9439	0.9019	0.9867	0.9702	0.9446	0.9956	0.8501	0.7336	0.982
	Alcohol Grams per Day	0.9998	0.9996	1.0001	0.998	0.9972	0.9987	1.0005	1	1.0009	1	0.9975	1.0024
	Diabetes Prevalence	1.0205	0.9719	1.0719	1.4835	1.2748	1.7302	1.0723	0.982	1.1656	0.7378	0.4451	1.2038
	Zero Education Proportion	1.0222	1.006	1.0389	1.0524	1.0004	1.1062	0.9231	0.8973	0.9497	0.8404	0.7142	0.9914
	Education Years per Capita	0.9988	0.9975	1.0001	0.9991	0.9953	1.003	0.9907	0.9885	0.9928	0.9778	0.966	0.9902
	Maternal Alcohol Consumption	0.9858	0.9671	1.0053	0.9974	0.9394	1.0609	0.9735	0.9394	1.0075	1.2291	1.0198	1.4832
	Mean Adult BMI	0.9976	0.9951	1	0.9952	0.9876	1.0029	0.9972	0.9927	1.0017	1.0114	0.9872	1.0365
	Obesity Prevalence	1.0697	1.0104	1.1326	1.1079	0.9235	1.3249	1.0287	0.9242	1.1419	0.8836	0.4975	1.5886
	Vitamin A Deficiency Prevalence	1.0436	1.0129	1.0743	1.0409	0.9516	1.1378	0.9733	0.9239	1.0253	2.3141	1.7482	3.0525
	**Rates of Change 1990-2020**	**Model: Blindness**			**Model: MSVI**			**Model: Mild**			**Model: Near Vision**		
**Sex**	**Covariate**	**OR**	**2.50%**	**97.50%**	**OR**	**2.50%**	**97.50%**	**OR**	**2.50%**	**97.50%**	**OR**	**2.50%**	**97.50%**
**MALE**	Smoking Prevalence Rate of Change	0.9883	0.93	1.0528	0.9951	0.9678	1.0232	0.9959	0.9717	1.02	1.0006	0.968	1.0349
	Alcohol Binge Drinker Proportion Rate of	1.0497	0.9027	1.2141	0.9708	0.909	1.0379	1.0592	1.0006	1.1237	1.0292	0.9516	1.1145
	Alcohol Grams per Day Rate of Change	1.6252	1.1732	2.2543	1.1182	0.9689	1.2898	0.9716	0.8613	1.1046	0.9751	0.8177	1.1626
	Diabetes Prevalence Rate of Change	1.038	0.971	1.1104	0.9662	0.938	0.995	0.987	0.9613	1.0135	0.9932	0.9584	1.0287
	Zero Education Proportion Rate of Chang	1.0309	0.9861	1.0789	1.0095	0.9898	1.0291	1.0133	0.9965	1.0309	1.0052	0.982	1.0291
	Education Years per Capita Rate of Change	0.9432	0.9161	0.9713	0.9778	0.9651	0.9904	0.9807	0.9704	0.992	0.9646	0.9498	0.9796
	Maternal Alcohol Consumption Rate of Ch	0.9789	0.2313	4.1197	0.9053	0.4847	1.7295	0.7268	0.4142	1.3019	0.8557	0.4001	1.8702
	Mean Adult BMI Rate of Change	0.5171	0.3101	0.8518	0.9418	0.7556	1.1807	1.0445	0.8599	1.2718	0.6833	0.5189	0.8946
	Obesity Prevalence Rate of Change	0.9922	0.9862	0.9982	1.0008	0.9983	1.0035	0.9955	0.9932	0.9979	0.998	0.9948	1.0012
	Vitamin A Deficiency Prevalence Rate of Ch	1.2502	1.1442	1.3681	1.0267	0.9864	1.0703	1.0633	1.0254	1.1026	1.0809	1.029	1.135
**FEMALE**	Smoking Prevalence Rate of Change	1.0429	0.9785	1.1133	1.0036	0.9795	1.0295	1.0025	0.9811	1.0243	1.0214	0.9921	1.0514
	Alcohol Binge Drinker Proportion Rate of	1.0175	0.9353	1.1086	1.0271	0.9936	1.0603	1.077	1.0463	1.1073	1.0283	0.9916	1.0684
	Alcohol Grams per Day Rate of Change	0.9968	0.7184	1.3917	0.9319	0.8201	1.0593	0.9249	0.8279	1.0301	0.9094	0.7874	1.0576
	Diabetes Prevalence Rate of Change	1.0466	0.9754	1.1222	0.9847	0.9567	1.0129	1.0112	0.9871	1.036	1.0093	0.978	1.043
	Zero Education Proportion Rate of Chang	1.1153	1.0456	1.1916	1.0116	0.9862	1.0385	1.0302	1.0078	1.0524	1.0148	0.9859	1.0452
	Education Years per Capita Rate of Change	0.9671	0.9523	0.9828	0.9866	0.9802	0.9928	0.9908	0.9857	0.996	0.9821	0.9753	0.9888
	Maternal Alcohol Consumption Rate of Ch	1.186	0.2386	6.0999	0.9881	0.5166	1.904	0.7589	0.4312	1.3325	0.7838	0.378	1.6352
	Mean Adult BMI Rate of Change	0.6238	0.3838	1.0118	0.9025	0.7397	1.1007	0.8281	0.7012	0.975	0.7368	0.598	0.9135
	Obesity Prevalence Rate of Change	0.9919	0.982	1.0015	1.001	0.9973	1.0048	0.9933	0.9902	0.9965	0.998	0.9938	1.0023
	Vitamin A Deficiency Prevalence Rate of Ch	1.2128	1.1005	1.3352	1.0095	0.9704	1.0505	1.0488	1.0153	1.0844	1.0929	1.0467	1.1406

Table S3. Global Risk Factors associated with age-standardised prevalence of blindness (BI), moderate and severe (MSVI), Mild and Near Vision impairments for 193 countries: Single and multiple variable odds ratios and 95% UI for **Age Specific Pooled.**

	**Univariable analysis: AGE-SPECIFIC (POOLED, NON-NESTED) ALL CAUSES**												
	**Prevalence 2020**	**Model: Blindness**			**Model: MSVI**			**Model: Mild**			**Model: Near Vision**		
**Sex**	**Covariate**	**OR**	**2.50%**	**97.50%**	**OR**	**2.50%**	**97.50%**	**OR**	**2.50%**	**97.50%**	**OR**	**2.50%**	**97.50%**
**MALE**	Smoking Prevalence (Age-specific)	0.9429	0.9358	0.9499	0.8638	0.8462	0.8807	0.8962	0.8855	0.907	0.8258	0.7969	0.8553
**FEMALE**	Smoking Prevalence (Age-specific)	0.894	0.8796	0.9091	0.7214	0.6945	0.7495	0.7917	0.7738	0.8098	0.5911	0.5532	0.6328
**MALE**	Secondhand Smoke	0.9195	0.9106	0.9283	0.7895	0.7687	0.8103	0.8478	0.8348	0.8606	0.6087	0.5808	0.6364
**FEMALE**	Secondhand Smoke	0.9264	0.9175	0.935	0.8055	0.7882	0.8235	0.8499	0.8389	0.8609	0.7014	0.6755	0.7284
**MALE**	Education Years Per Capita	0.9932	0.993	0.9935	0.9821	0.9813	0.9828	0.9892	0.9888	0.9896	0.9696	0.9683	0.9709
**FEMALE**	Education Years Per Capita	0.9935	0.9932	0.9938	0.9837	0.983	0.9844	0.9896	0.9892	0.9899	0.9711	0.97	0.9723
**MALE**	Cumulative Cigarettes (15Y)	1	1	1	1	1	1	1	1	1	1	1	1
**FEMALE**	Cumulative Cigarettes (15Y)	1	1	1	1	1	1	1	1	1	1	1	1
**MALE**	Cumulative Cigarettes (20Y)	1	1	1	1	1	1	1	1	1	1	1	1
**FEMALE**	Cumulative Cigarettes (20Y)	1	1	1	1	1	1	1	1	1	1	1	1
**MALE**	Education Gini Index	1.125	1.1202	1.1298	1.3422	1.3253	1.3579	1.1846	1.1769	1.1921	1.6168	1.5829	1.6509
**FEMALE**	Education Gini Index	1.1119	1.1073	1.1165	1.2811	1.2682	1.294	1.164	1.1571	1.1708	1.5377	1.5102	1.5661
	**Rates of Change 1990-2020**	**Model: Blindness**			**Model: MSVI**			**Model: Mild**			**Model: Near Vision**		
**Sex**	**Covariate**	**OR**	**2.50%**	**97.50%**	**OR**	**2.50%**	**97.50%**	**OR**	**2.50%**	**97.50%**	**OR**	**2.50%**	**97.50%**
**MALE**	Smoking Prevalence (Age-specific) Rate of Change	0.9414	0.9262	0.9572	0.981	0.9759	0.9861	0.9739	0.9688	0.979	0.971	0.962	0.98
**FEMALE**	Smoking Prevalence (Age-specific) Rate of Change	1.0165	1.0028	1.0305	0.9963	0.9918	1.0008	0.9932	0.9883	0.9979	1.0056	0.9973	1.0138
**MALE**	Secondhand Smoke Rate of Change	0.877	0.8492	0.9062	0.9658	0.9556	0.9761	0.9377	0.9284	0.9475	0.9367	0.9194	0.9551
**FEMALE**	Secondhand Smoke Rate of Change	0.8931	0.8707	0.9166	0.9366	0.9288	0.9448	0.9185	0.9106	0.9265	0.9237	0.9097	0.9385
**MALE**	Education Years Per Capita Rate of Change	0.9615	0.9586	0.9645	0.991	0.99	0.992	0.9884	0.9875	0.9894	0.9827	0.9809	0.9845
**FEMALE**	Education Years Per Capita Rate of Change	0.9811	0.9797	0.9826	0.9947	0.9942	0.9952	0.9944	0.9938	0.9949	0.9905	0.9897	0.9914
**MALE**	Cumulative Cigarettes (15Y) Rate of Change	1.0027	0.9908	1.0146	0.9937	0.9899	0.9975	0.9947	0.9906	0.9986	1.0054	0.9979	1.0129
**FEMALE**	Cumulative Cigarettes (15Y) Rate of Change	1.0217	1.0113	1.0324	0.9987	0.9952	1.0023	0.998	0.9943	1.0018	1.0129	1.0066	1.0192
**MALE**	Cumulative Cigarettes (20Y) Rate of Change	1.0047	0.9928	1.0174	0.9934	0.9893	0.9974	0.9941	0.9901	0.9981	1.0037	0.9964	1.0112
**FEMALE**	Cumulative Cigarettes (20Y) Rate of Change	1.0198	1.0092	1.0307	0.998	0.9944	1.0015	0.9969	0.9932	1.0006	1.0106	1.0039	1.0171
**MALE**	Education Gini Index Rate of Change	1.1595	1.1314	1.1889	1.0133	1.0057	1.0216	1.0471	1.0389	1.0555	1.01	0.9948	1.0255
**FEMALE**	Education Gini Index Rate of Change	1.052	1.0315	1.0729	0.9934	0.9867	1.0001	1.0129	1.0061	1.0198	0.9797	0.9677	0.991
	**Multivariable analysis: AGE-SPECIFIC (NON-NESTED) ALL CAUSES**												
	**Prevalence 2020**	**Model: Blindness**			**Model: MSVI**			**Model: Mild**			**Model: Near Vision**		
**Sex**	**Covariate**	**OR**	**2.50%**	**97.50%**	**OR**	**2.50%**	**97.50%**	**OR**	**2.50%**	**97.50%**	**OR**	**2.50%**	**97.50%**
**MALE**	Smoking Prevalence (Age-specific)	0.9518	0.9443	0.9595	0.8519	0.8324	0.8728	0.8989	0.8877	0.9103	0.7789	0.7493	0.8101
	Secondhand Smoke	0.9732	0.9652	0.9813	0.9276	0.9073	0.9494	0.921	0.9094	0.9325	0.8145	0.7823	0.8472
	Education Years Per Capita	1.0002	0.9994	1.001	0.9908	0.9887	0.9929	0.9908	0.9896	0.9919	0.9746	0.971	0.9784
	Cumulative Cigarettes (15Y)	1	1	1	1	1	1	1	1	1	1	1	1
	Cumulative Cigarettes (20Y)	1	1	1	1	1	1	1	1	1	1	1	1
	Education Gini Index	1.1206	1.1065	1.1346	1.146	1.1072	1.1883	1.0071	0.9886	1.026	1.0596	0.9968	1.1275
**FEMALE**	Smoking Prevalence (Age-specific)	0.9868	0.9642	1.0102	0.9021	0.8531	0.954	0.9502	0.9209	0.9793	0.8706	0.7876	0.963
	Secondhand Smoke	0.9628	0.9552	0.9702	0.8914	0.8748	0.9087	0.9015	0.8922	0.9109	0.8472	0.8184	0.8762
	Education Years Per Capita	1.0006	0.9997	1.0015	0.9901	0.988	0.9922	0.9905	0.9894	0.9917	0.9749	0.9712	0.9785
	Cumulative Cigarettes (15Y)	1	1	1	1	1	1	1	1	1	1	1	1
	Cumulative Cigarettes (20Y)	1	1	1	1	1	1	1	1	1	1	1	1
	Education Gini Index	1.1159	1.102	1.1309	1.0931	1.0584	1.1274	0.9984	0.9806	1.0157	1.0531	0.9952	1.1154
	**Rates of Change 1990-2020**	**Model: Blindness**			**Model: MSVI**			**Model: Mild**			**Model: Near Vision**		
**Sex**	**Covariate**	**OR**	**2.50%**	**97.50%**	**OR**	**2.50%**	**97.50%**	**OR**	**2.50%**	**97.50%**	**OR**	**2.50%**	**97.50%**
**MALE**	Smoking Prevalence (Age-specific) Rate of Change	0.8872	0.8617	0.915	0.972	0.9625	0.9813	0.9538	0.9448	0.9633	0.8958	0.8788	0.9129
	Secondhand Smoke Rate of Change	0.9636	0.9331	0.9954	0.9897	0.9789	1.0003	0.9733	0.9627	0.9841	0.9914	0.9717	1.012
	Education Years Per Capita Rate of Change	0.965	0.9621	0.9678	0.9917	0.9907	0.9927	0.9899	0.989	0.9909	0.9843	0.9824	0.9861
	Cumulative Cigarettes (15Y) Rate of Change	1.1023	1.0167	1.1986	1.048	1.0192	1.077	1.0914	1.0625	1.1205	1.2631	1.1987	1.3296
	Cumulative Cigarettes (20Y) Rate of Change	0.9925	0.9202	1.0689	0.9668	0.9427	0.9916	0.942	0.9196	0.9652	0.8511	0.8126	0.8939
	Education Gini Index Rate of Change	1.1508	1.1241	1.1791	1.0127	1.0045	1.0208	1.0391	1.0311	1.0472	1.005	0.9899	1.0198
**FEMALE**	Smoking Prevalence (Age-specific) Rate of Change	1.0042	0.9852	1.0236	0.9985	0.9919	1.005	0.9951	0.9884	1.0018	0.9886	0.9766	1.0002
	Secondhand Smoke Rate of Change	0.976	0.9497	1.0036	0.9556	0.947	0.9645	0.9357	0.9267	0.9448	0.9505	0.9341	0.9673
	Education Years Per Capita Rate of Change	0.9808	0.9793	0.9823	0.9956	0.9951	0.9961	0.9956	0.9951	0.9961	0.9918	0.9909	0.9928
	Cumulative Cigarettes (15Y) Rate of Change	1.0207	0.9616	1.0855	1.0172	0.9964	1.0398	1.036	1.0134	1.0585	1.0863	1.0469	1.1259
	Cumulative Cigarettes (20Y) Rate of Change	0.9997	0.9447	1.0586	0.9861	0.966	1.0054	0.9731	0.9536	0.9933	0.94	0.9081	0.9741
	Education Gini Index Rate of Change	1.1076	1.0869	1.1287	1.0032	0.9965	1.0099	1.0223	1.0155	1.0291	1.0074	0.9958	1.0194

Appendix

Table S4. Significant Risk Factors associated with age-standardised prevalence of moderate and severe vision impairment **(MSVI)** for 193 countries: Single and multiple variable analysis, global estimated odds ratios.

Prevalence	MSVI, Single Variable		MSVI, Multiple Variable	
**Covariate, All Persons**	**OR (95% UI)**		**OR (95% UI)**	
Smoking (All)	0.9224 (0.8584,0.9906)		1.1509 (1.0617,1.2494)	
Outdoor Pollution	1.0012 (1.0009,1.0014)		1.0009 (1.0006,1.0012)	
Indoor Pollution	1.0474 (1.0312,1.0633)		0.9912 (0.9652,1.0191)	
Sanitation (Proportion)	0.9438 (0.9224,0.9666)		1.0540 (1.0156,1.0951)	
Maternal Care & Immunisation	0.9837 (0.9794,0.9878)		1 (0.9929,1.0072)	
Healthcare Access & Quality index	0.9990 (0.9988,0.9992)		0.9993 (0.9988,0.9999)	
Ophthalmologists PMP	0.9995 (0.9994,0.9996)		0.9998 (0.9997,1)	
Socio-Demographic Index	0.8852 (0.8602,0.9110)		0.9380 (0.8701,1.0113)	
**Prevalence**	**OR (95% UI)**		**OR (95% UI)**	
**Covariate, by Sex**	**Female**	**Male**	**Female**	**Male**
Alcohol Binge Drinker (Proportion)	0.8548 (0.8273,0.8829)	0.8797 (0.8597,0.9004)	0.9439 (0.9019,0.9867)	0.9550 (0.9236,0.9867)
Maternal Alcohol Consumption	0.8186 (0.7760,0.8635)	0.8196 (0.7818,0.8590)	0.9974 (0.9394,1.0609)	0.9942 (0.9408,1.0484)
Vitamin A Deficiency	1.2201 (1.1187,1.3314)	1.2469 (1.1553,1.3433)	1.0409 (0.9516,1.1378)	1.0044 (0.9255,1.0893)
Education (Years Per Capita)	0.9932 (0.9917,0.9947)	0.9918 (0.9902,0.9935)	0.9991 (0.9953,1.0030)	0.9952 (0.9919,0.9986)
Zero Education (Proportion)	1.1034 (1.0764,1.1295)	1.1290 (1.0922,1.1694)	1.0524 (1.0004,1.1062)	0.9857 (0.9349,1.0381)
Smoking (F/M)	0.7895 (0.7442,0.8382)	1.0438 (0.9922,1.0995)	0.9148 (0.8537,0.9790)	1.0604 (1.0196,1.1013)
Second-hand Smoke (Age-Specific, Pooled)	0.8055 (0.7882,0.8235)	0.7895 (0.7687,0.8103)	0.8914 (0.8748,0.9087)	0.9276 (0.9073,0.9494)
Edication Gini (Age-Specific, Pooled)	1.2811 (1.2682,1.2940)	1.3422 (1.3253,1.3579)	1.0931 (1.0584,1.1274)	1.1460 (1.1072,1.1883)

PMP= Per Million Population

Appendix

Figure S1 GBD Super-regional breakdowns of single-variable associations for age-standardised **blindness and MSVI** and selected risk factors 2020 in older persons (>50yrs), where the size of data points is weighted by each country's population to highlight populous countries of note. **Both sexes combined.**

Gray shading refers to the estimate and confidence bands of the global, non-super-regional coefficient

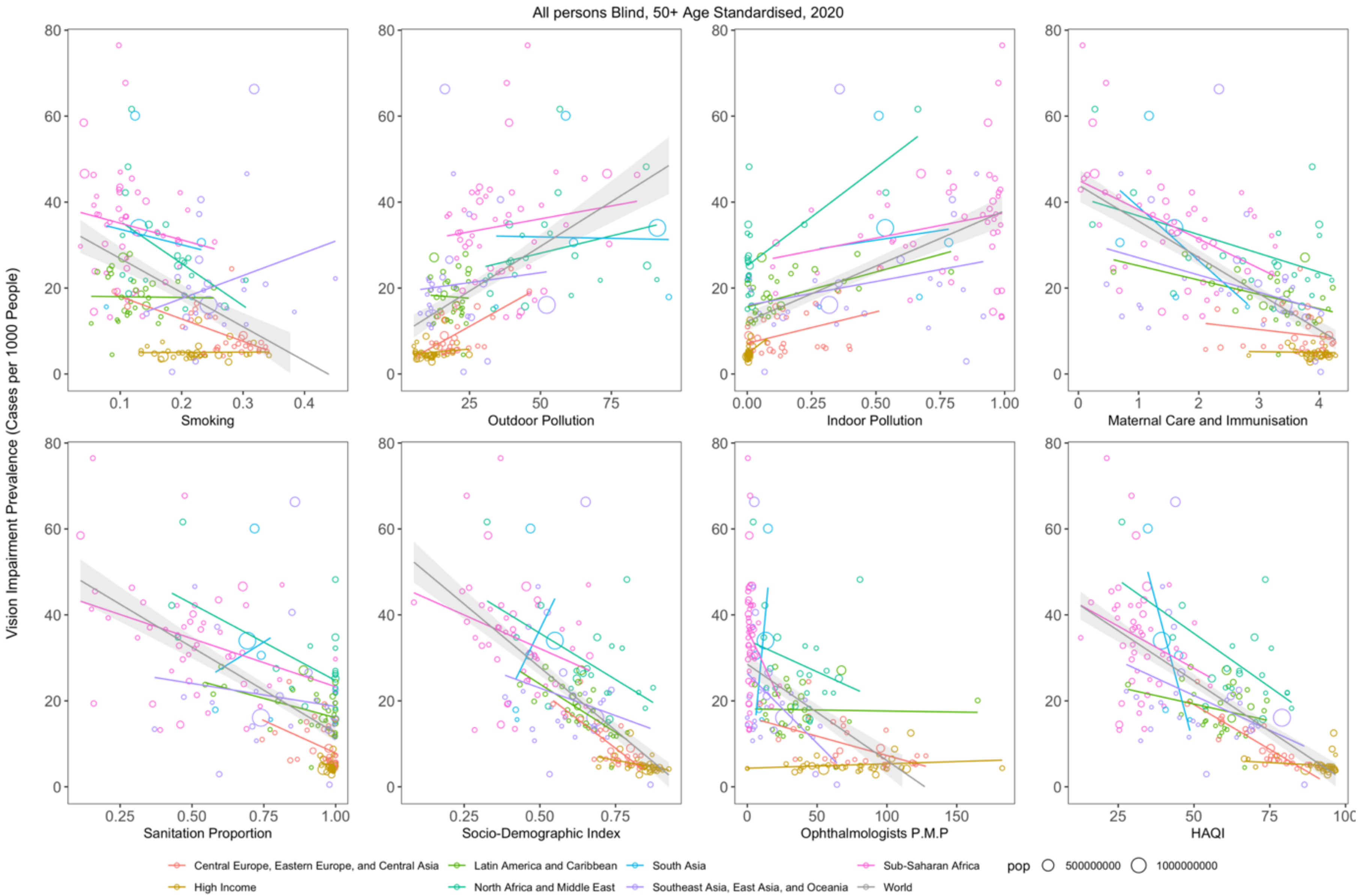

All persons Blind, 50+ Age Standardised, 2020
Vision Impairment Prevalence (Cases per 1000 People)
Smoking
Outdoor Pollution
Indoor Pollution
Maternal Care and Immunisation
Sanitation Proportion
Socio-Demographic Index
Ophthalmologists P.M.P
HAQI
Central Europe, Eastern Europe, and Central Asia
High Income
Latin America and Caribbean
North Africa and Middle East
South Asia
Southeast Asia, East Asia, and Oceania
Sub-Saharan Africa
World
pop
500000000
1000000000

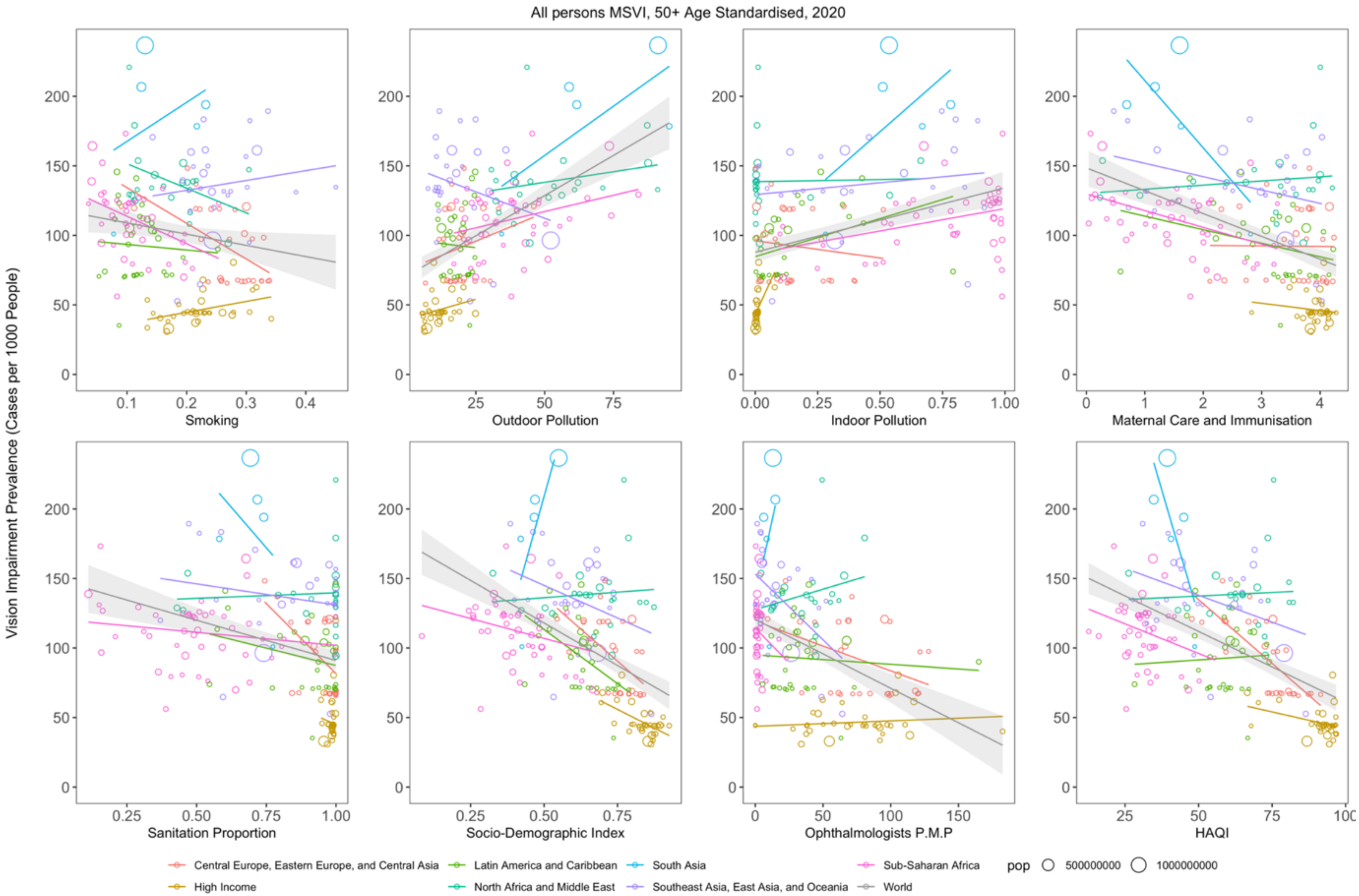

All persons MSVI, 50+ Age Standardised, 2020
Vision Impairment Prevalence (Cases per 1000 People)
Smoking
Outdoor Pollution
Indoor Pollution
Maternal Care and Immunisation
Sanitation Proportion
Socio-Demographic Index
Ophthalmologists P.M.P
HAQI
Central Europe, Eastern Europe, and Central Asia
High Income
Latin America and Caribbean
North Africa and Middle East
South Asia
Southeast Asia, East Asia, and Oceania
Sub-Saharan Africa
World
pop
500000000
1000000000

Appendix

Figure S2. GBD Super-regional breakdowns of single-variable associations for age-standardised **blindness and MSVI** and selected risk factors 2020 in older persons (>50yrs), where the size of data points is weighted by each country's population to highlight populous countries of note. **Disaggregated by sex.**

Gray shading refers to the estimate and confidence bands of the global, non-super-regional coefficient

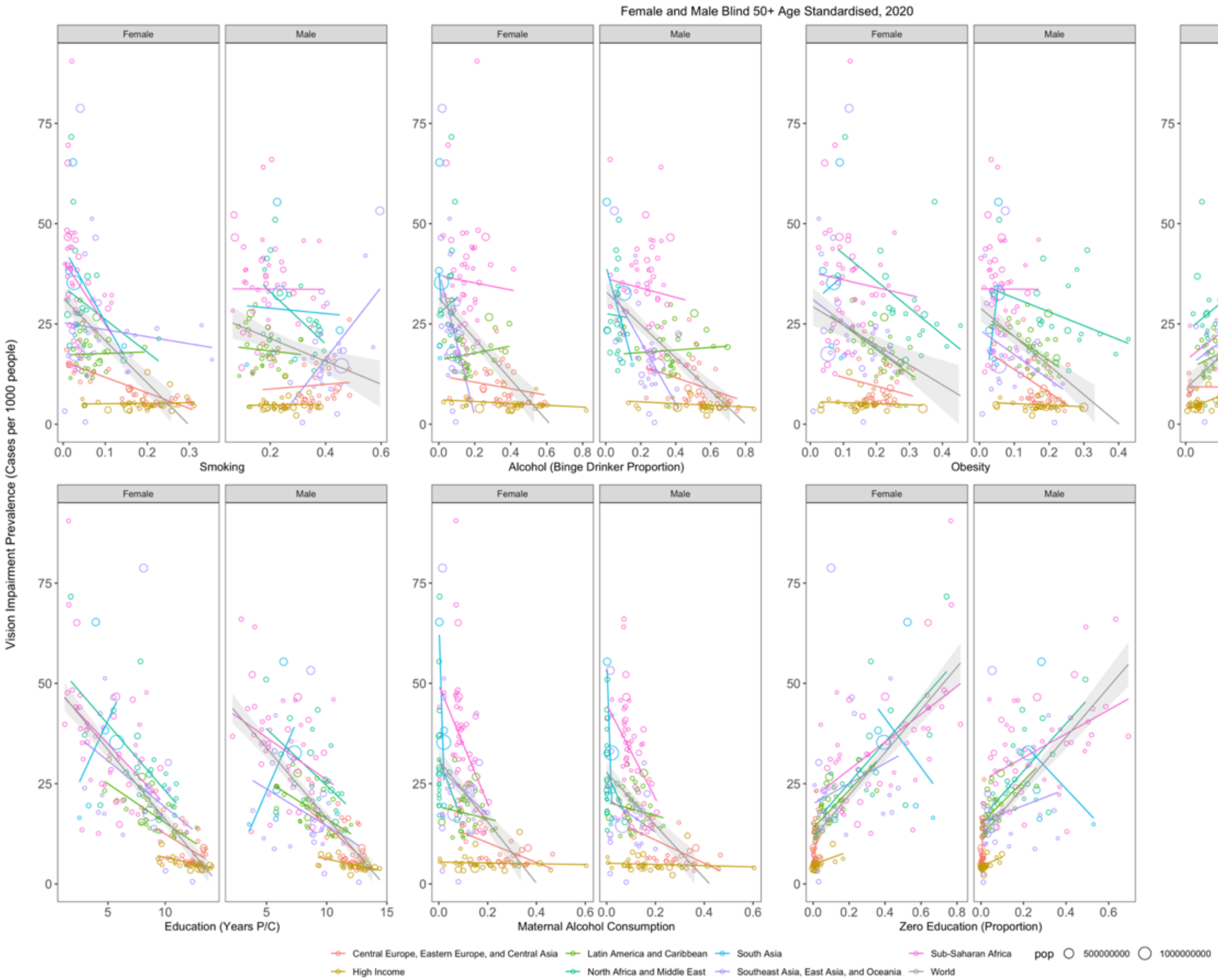
Female and Male Blind 50+ Age Standardised, 2020
Female
Male
Vision Impairment Prevalence (Cases per 1000 people)
Smoking
Alcohol (Binge Drinker Proportion)
Obesity
Vit A Deficiency
Education (Years P/C)
Maternal Alcohol Consumption
Zero Education (Proportion)
Central Europe, Eastern Europe, and Central Asia
High Income
Latin America and Caribbean
North Africa and Middle East
South Asia
Southeast Asia, East Asia, and Oceania
Sub-Saharan Africa
World
pop
500000000
1000000000

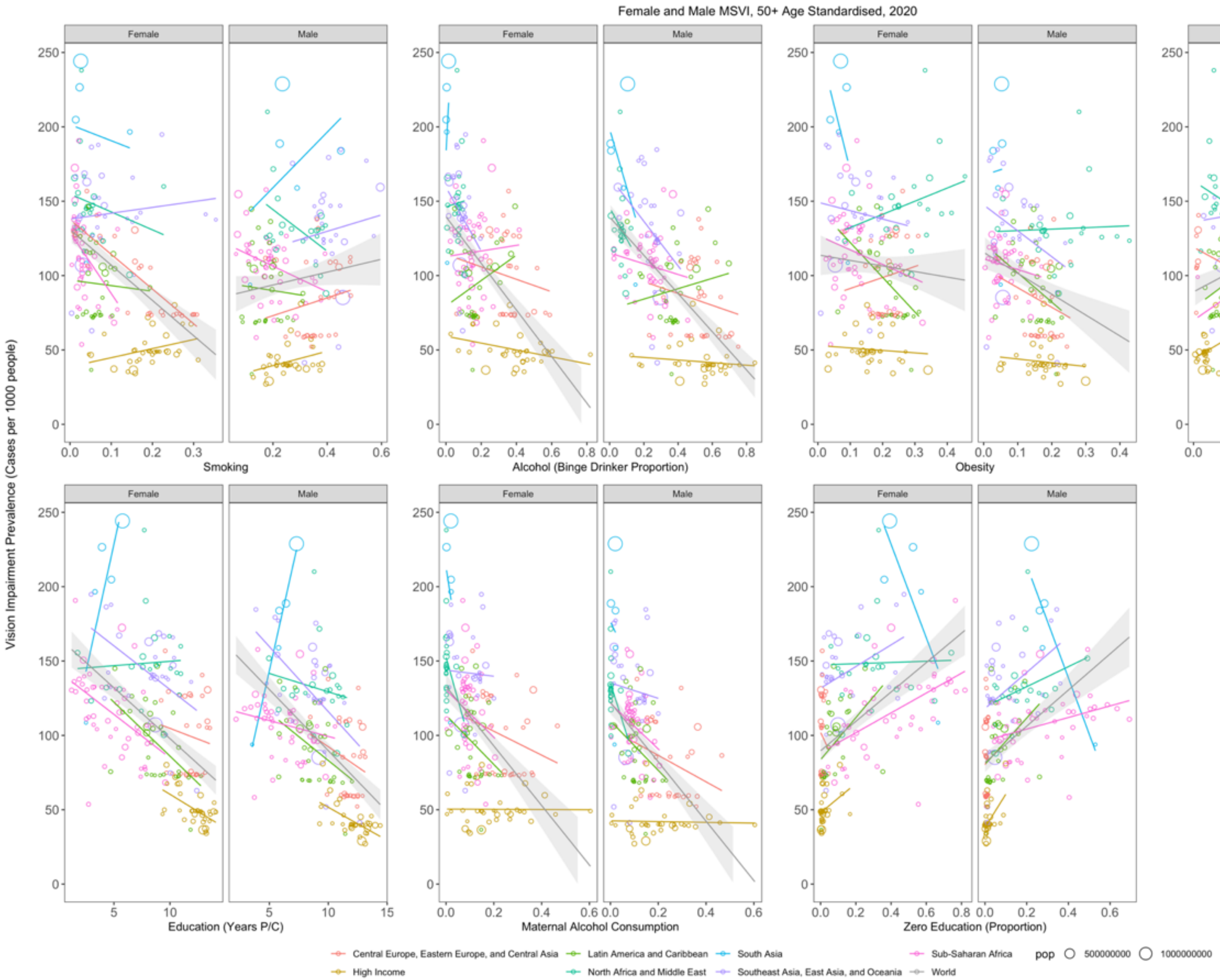

Female and Male MSVI, 50+ Age Standardised, 2020
Vision Impairment Prevalence (Cases per 1000 people)
Female
Male
Smoking
Alcohol (Binge Drinker Proportion)
Obesity
Vit A Deficiency
Education (Years P/C)
Maternal Alcohol Consumption
Zero Education (Proportion)
Central Europe, Eastern Europe, and Central Asia
High Income
Latin America and Caribbean
North Africa and Middle East
South Asia
Southeast Asia, East Asia, and Oceania
Sub-Saharan Africa
World
pop
500000000
1000000000

Appendix

Figure S3. Global Risk Factors for % rates of change between 1990 and 2020 in prevalence of blindness (BI), moderate and severe (MSVI), Mild and Near Vision impairments for 193 countries: Single and multiple variable odds ratios and 95% UI for **Females and Males.**

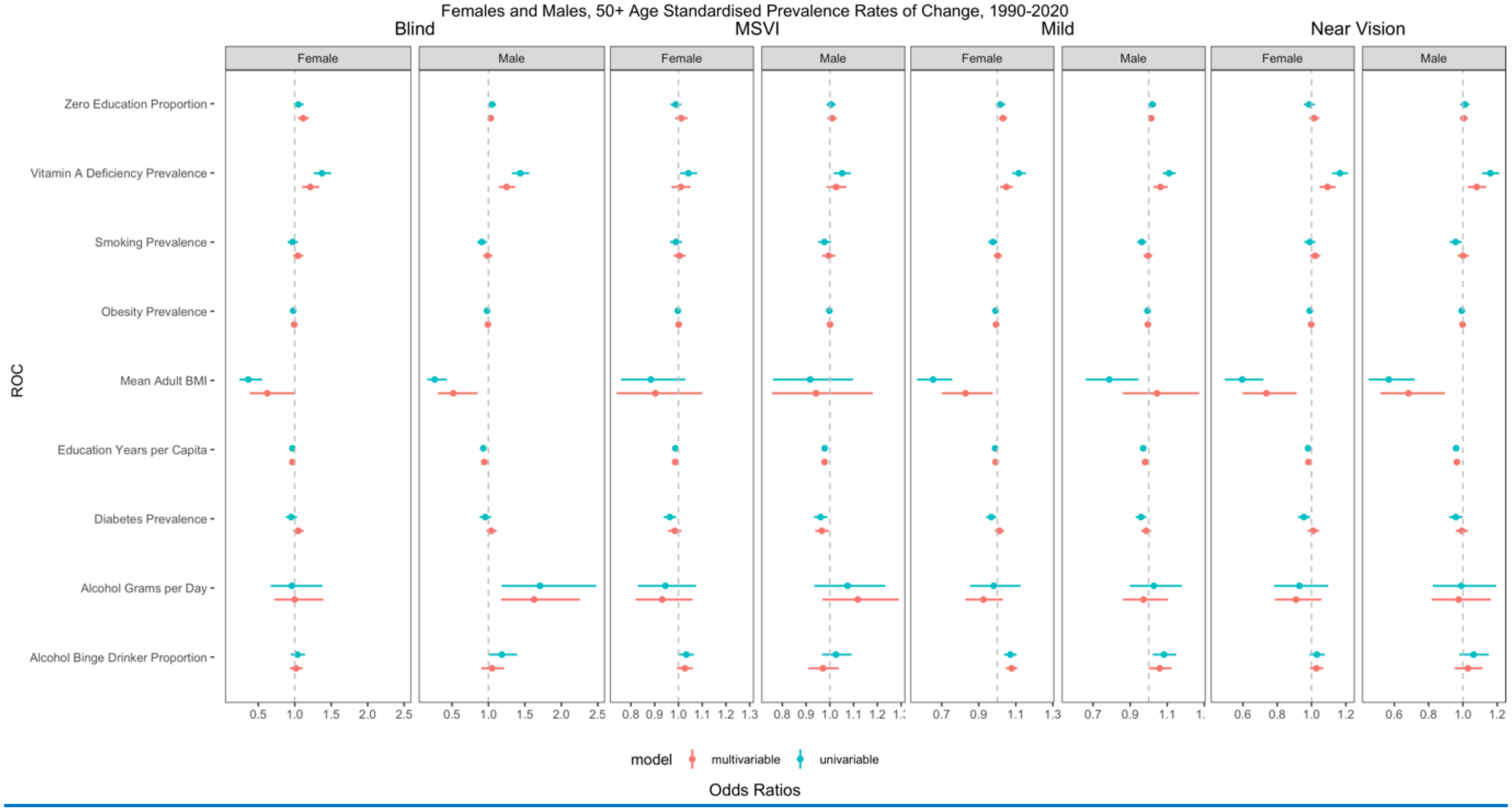

Appendix. Figure S4. GBD Super-regional breakdowns of single-variable associations for % rate of change in age-standardised **blindness and MSVI** and selected risk factors in older persons (>50yrs), where the size of data points is weighted by each country's population to highlight populous countries of note. **Both sexes combined.**

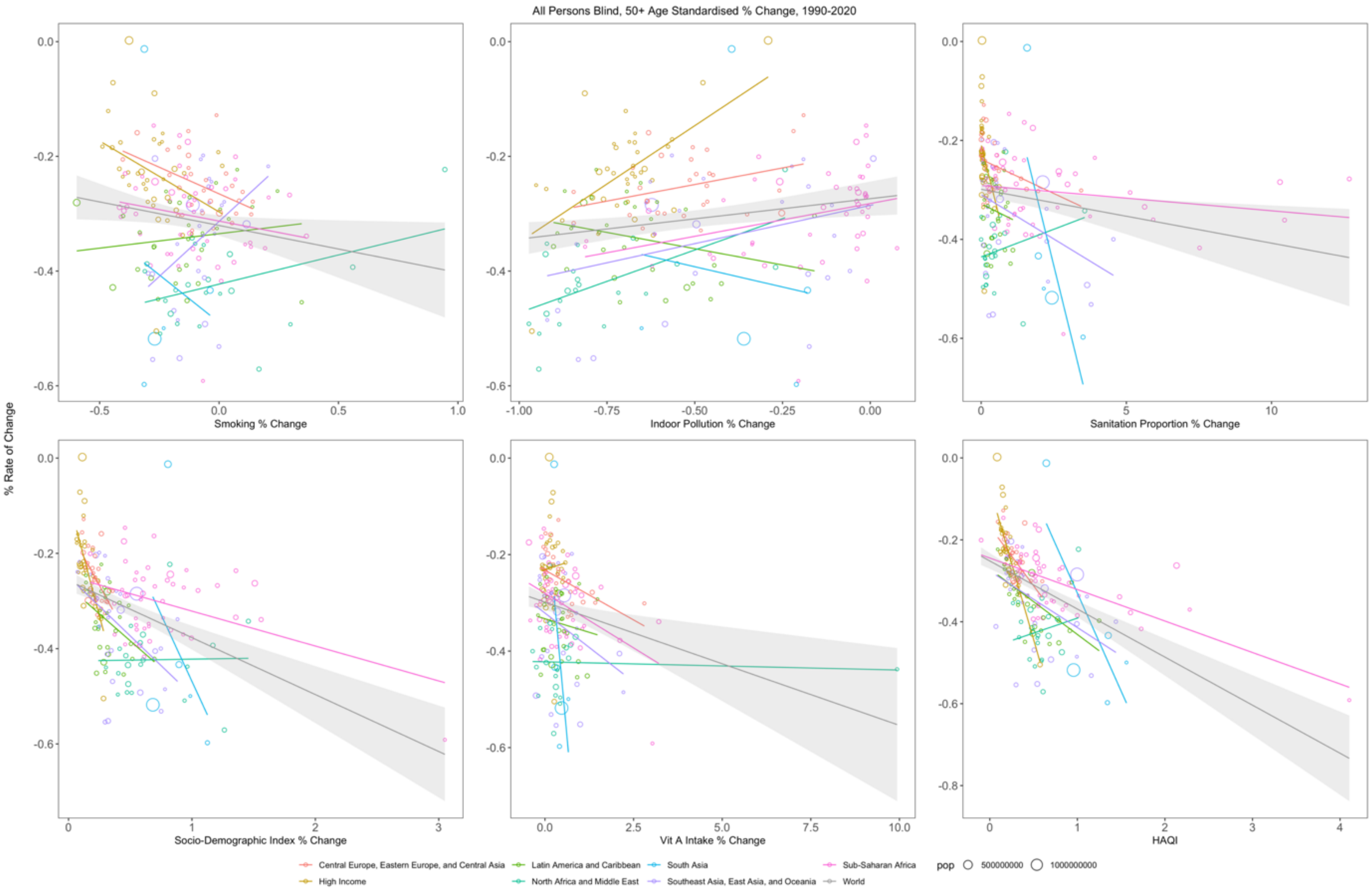

Gray shading refers to the estimate and confidence bands of the global, non-super-regional coefficient

Appendix

Figure S5. GBD Super-regional breakdowns of single-variable associations for % rate of change in age-standardised **blindness and MSVI** and selected risk factors in older persons (>50yrs), where the size of data points is weighted by each country's population to highlight populous countries of note. **Disaggregated by sex.**

Gray shading refers to the estimate and confidence bands of the global, non-super-regional coefficient

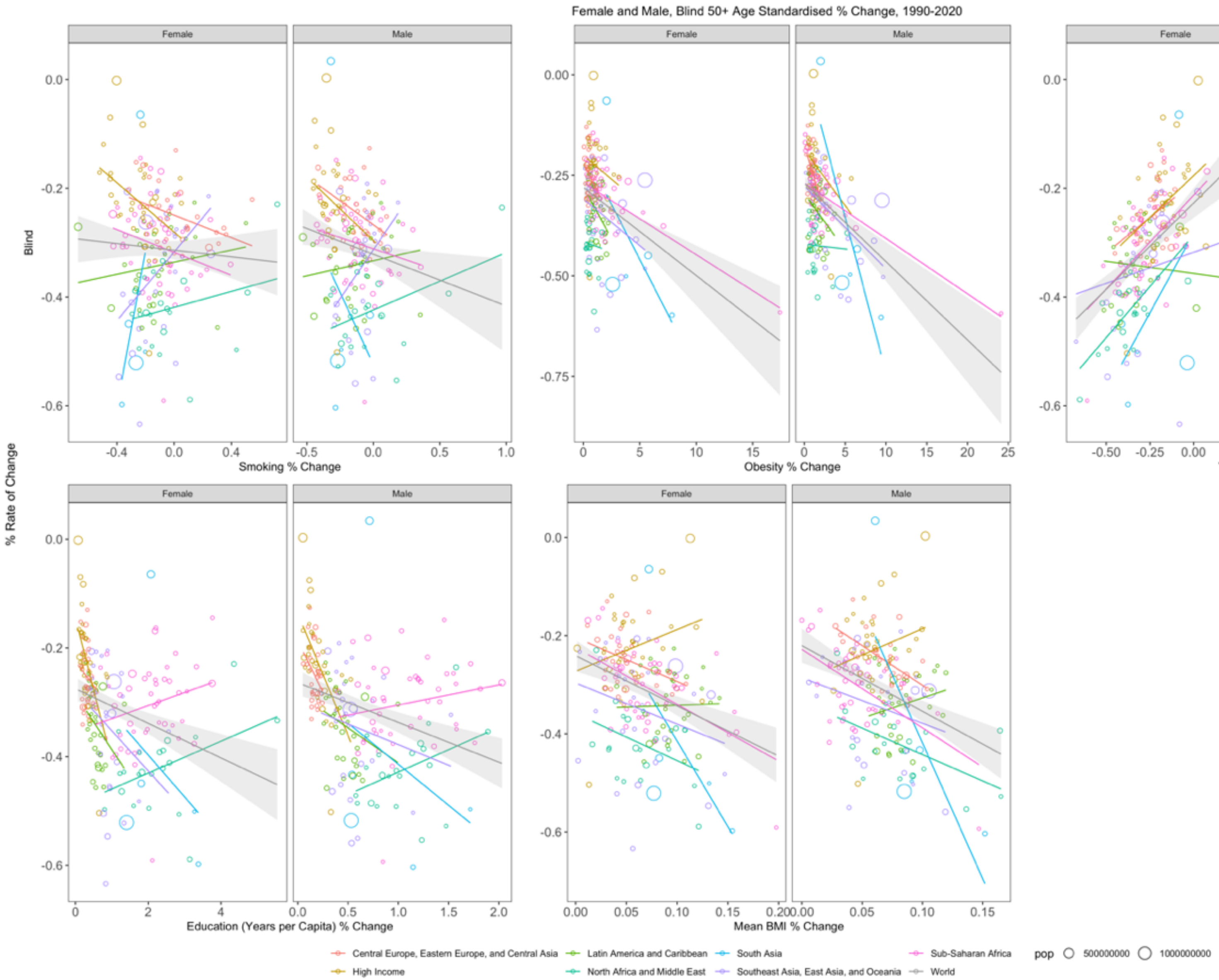

Female and Male, Blind 50+ Age Standardised % Change, 1990-2020
Female
Male
Blind
% Rate of Change
Smoking % Change
Obesity % Change
Vit A Deficiency % Change
Education (Years per Capita) % Change
Mean BMI % Change
Central Europe, Eastern Europe, and Central Asia
High Income
Latin America and Caribbean
North Africa and Middle East
South Asia
Southeast Asia, East Asia, and Oceania
Sub-Saharan Africa
World
pop
500000000
1000000000

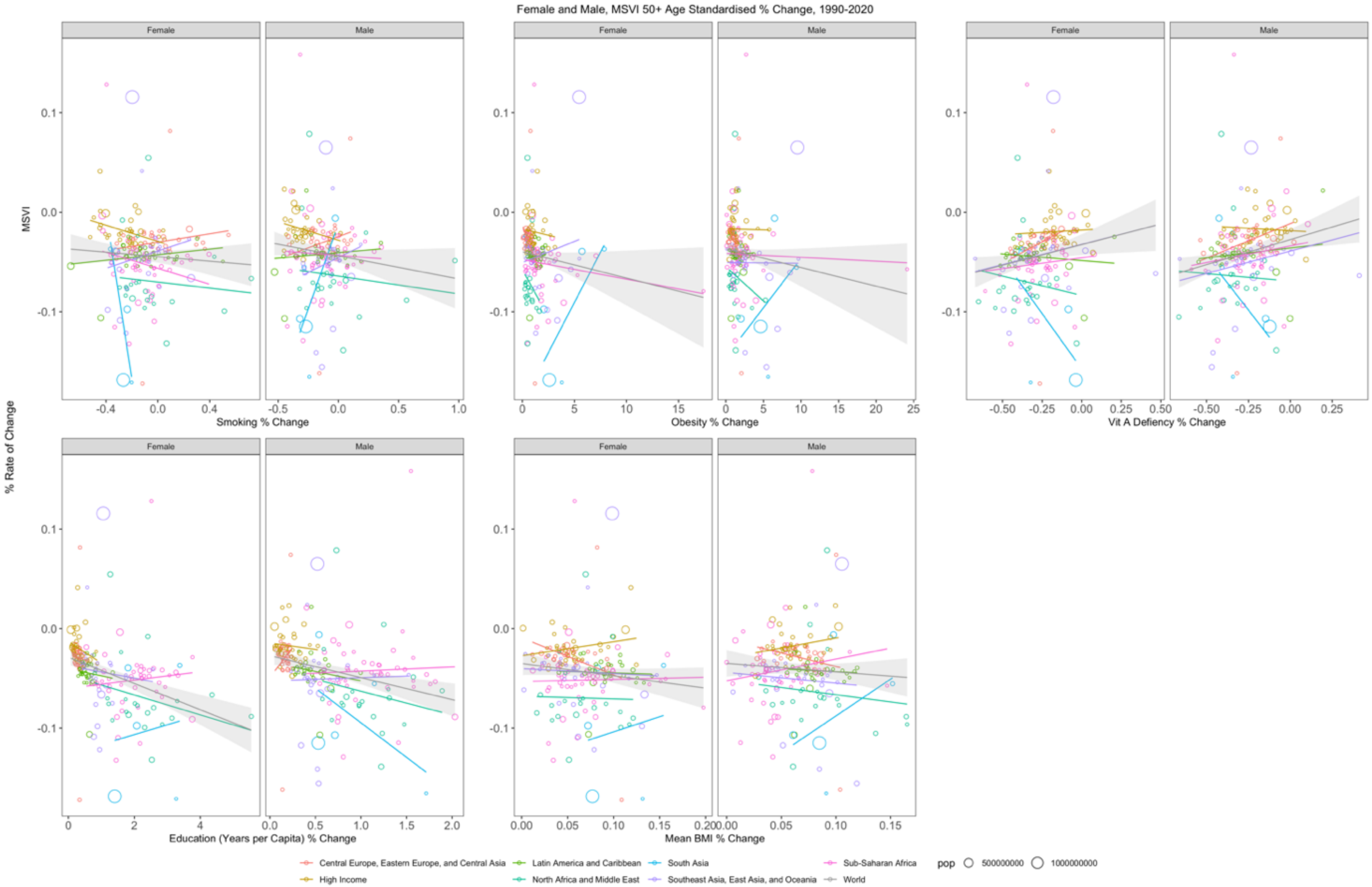

Female and Male, MSVI 50+ Age Standardised % Change, 1990-2020
Female
Male
MSVI
% Rate of Change
Smoking % Change
Obesity % Change
Vit A Defiency % Change
Education (Years per Capita) % Change
Mean BMI % Change
Central Europe, Eastern Europe, and Central Asia
High Income
Latin America and Caribbean
North Africa and Middle East
South Asia
Southeast Asia, East Asia, and Oceania
Sub-Saharan Africa
World
pop
500000000
1000000000

Appendix

Figure S6. Multilevel Risk Factors for prevalence in 2020 of **blindness** (BI) for age-specific Smoking, Second-hand Smoke, Current Drinkers, and Education Gini. All representative adult 5-year age groups, population-weighted, univariable analysis.

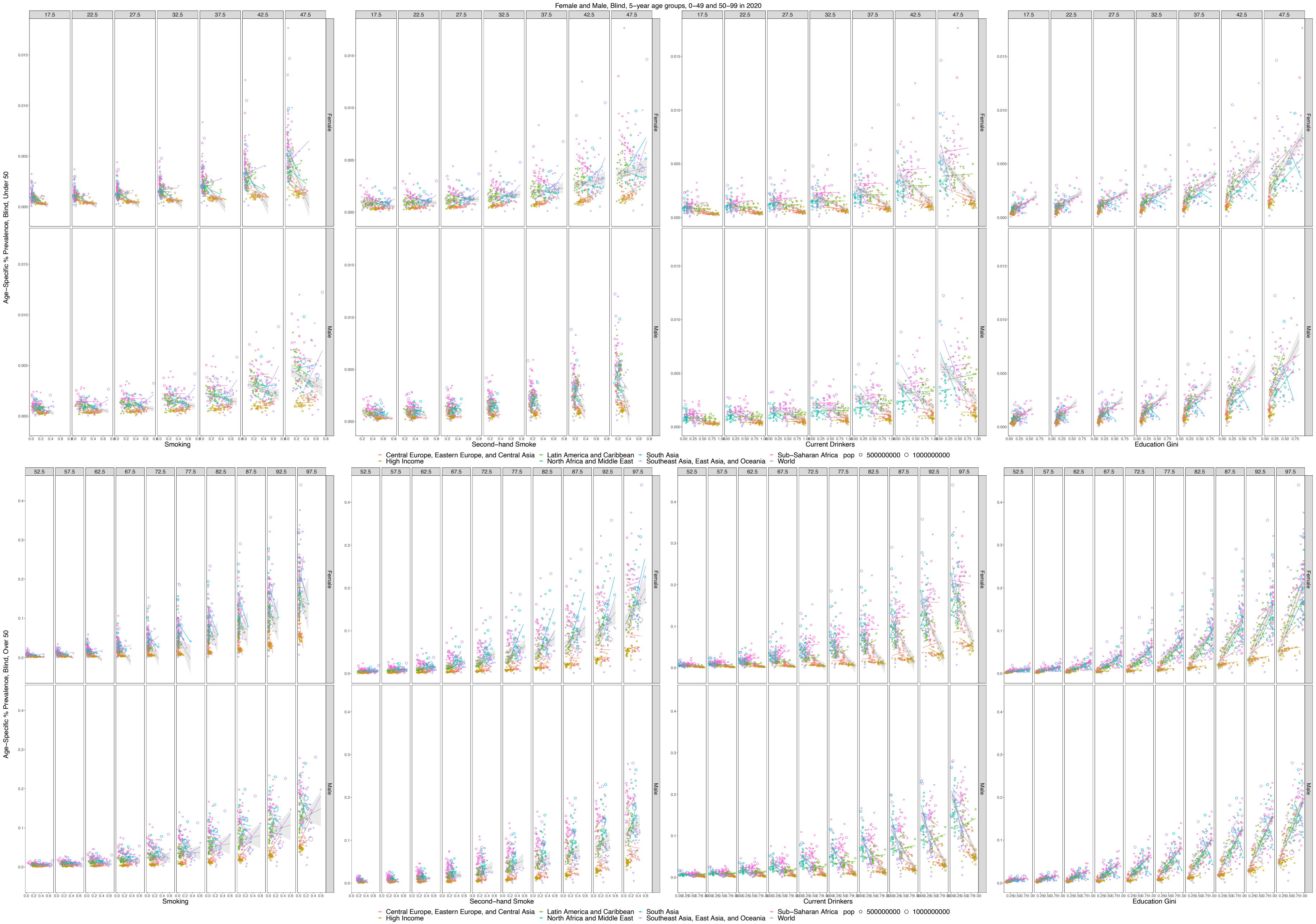
Female and Male, Blind, 5-year age groups, 0–49 and 50–99 in 2020
Age-Specific % Prevalence, Blind, Under 50
Age-Specific % Prevalence, Blind, Over 50
Female
Male
Smoking
Second-hand Smoke
Current Drinkers
Education Gini
Central Europe, Eastern Europe, and Central Asia
High Income
Latin America and Caribbean
North Africa and Middle East
South Asia
Southeast Asia, East Asia, and Oceania
Sub-Saharan Africa
World
pop
500000000
1000000000

Gray shading refers to the estimate and confidence bands of the global, non-super-regional coefficient

Appendix

Figure S7. Multilevel Risk Factors for prevalence in 2020 of **moderate and severe vision impairment** (MSVI) for age-specific Smoking, Second-hand Smoke, Current Drinkers, and Education Gini. All representative adult 5-year age groups, population-weighted, univariable analysis.

Gray shading refers to the estimate and confidence bands of the global, non-super-regional coefficient

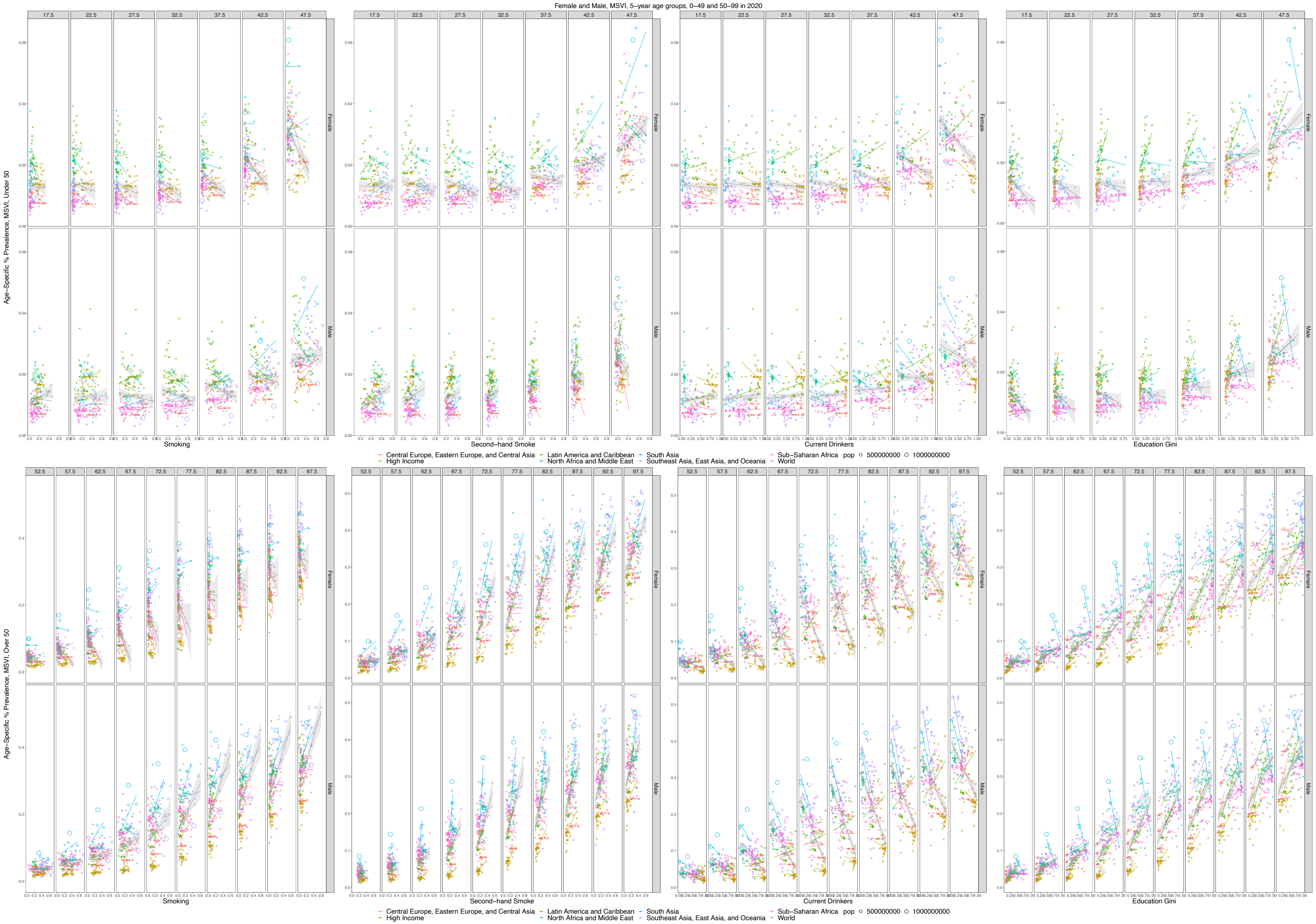
Female and Male, MSVI, 5-year age groups, 0-49 and 50-99 in 2020
Age-Specific % Prevalence, MSVI, Under 50
Age-Specific % Prevalence, MSVI, Over 50
Female
Male
Smoking
Second-hand Smoke
Current Drinkers
Education Gini
Central Europe, Eastern Europe, and Central Asia
High Income
Latin America and Caribbean
North Africa and Middle East
South Asia
Southeast Asia, East Asia, and Oceania
Sub-Saharan Africa
World
pop
500000000
1000000000

Appendix

Figure S8. Global and super-regional regressions for female:male sex ratios in 1990-2020 and % rate of change in prevalence and sex ratios of **blindness** (BI).

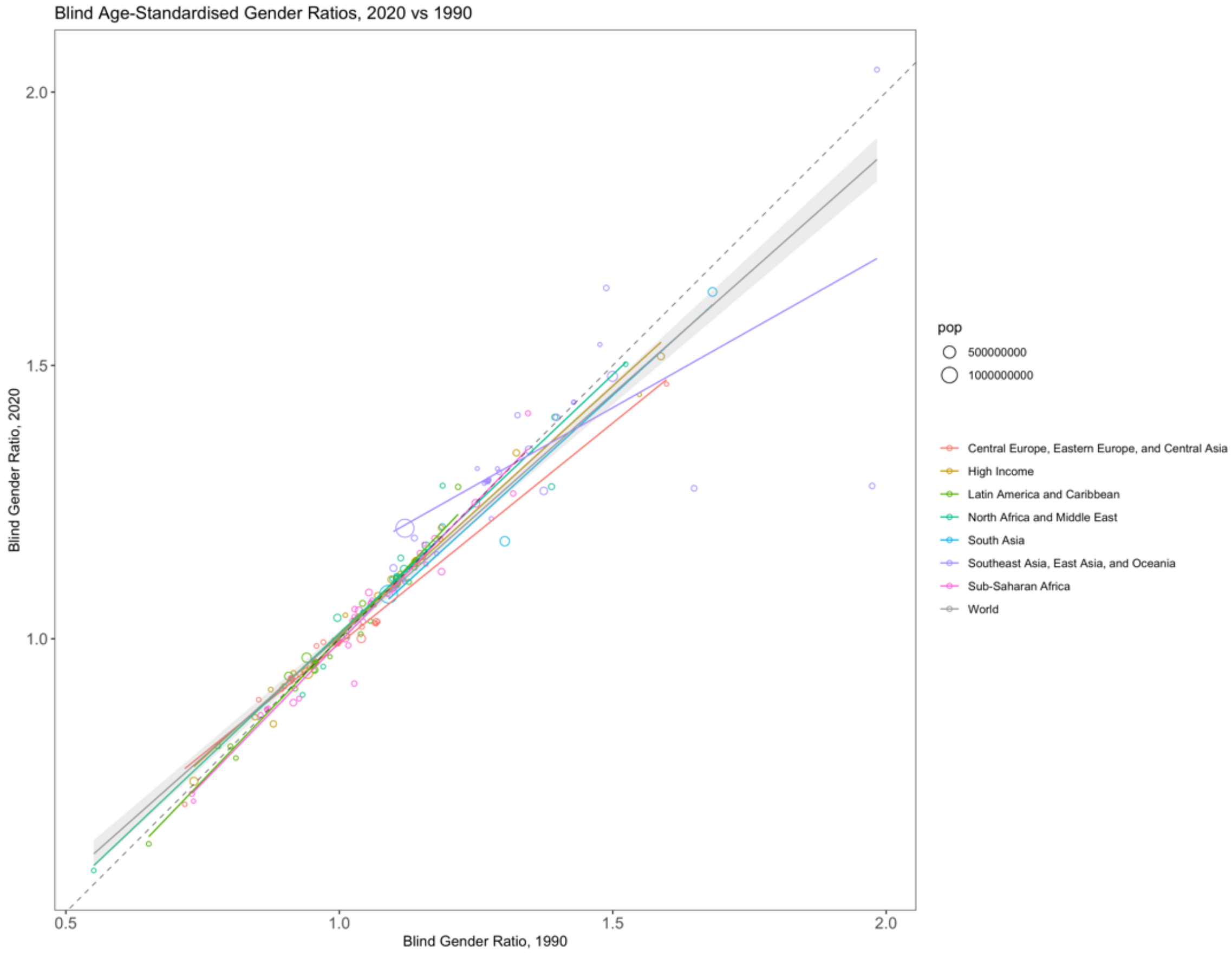

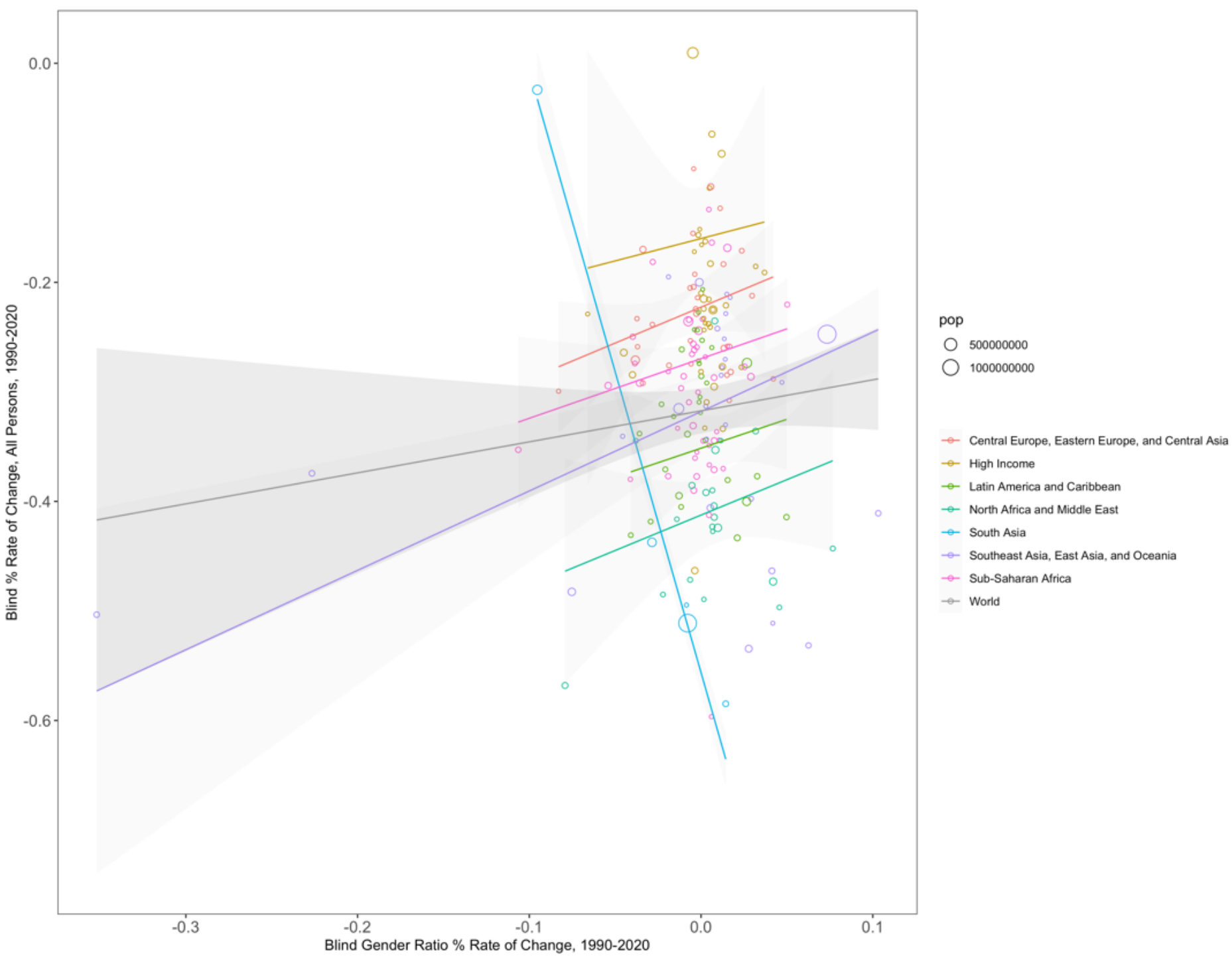

Gray shading refers to the estimate and confidence bands of the global, non-super-regional coefficient

Appendix

Figure S9. Global and super-regional regressions for female:male sex ratios in 1990-2020 and % rate of change in prevalence and sex ratios of **moderate and severe vision impairment** (MSVI).

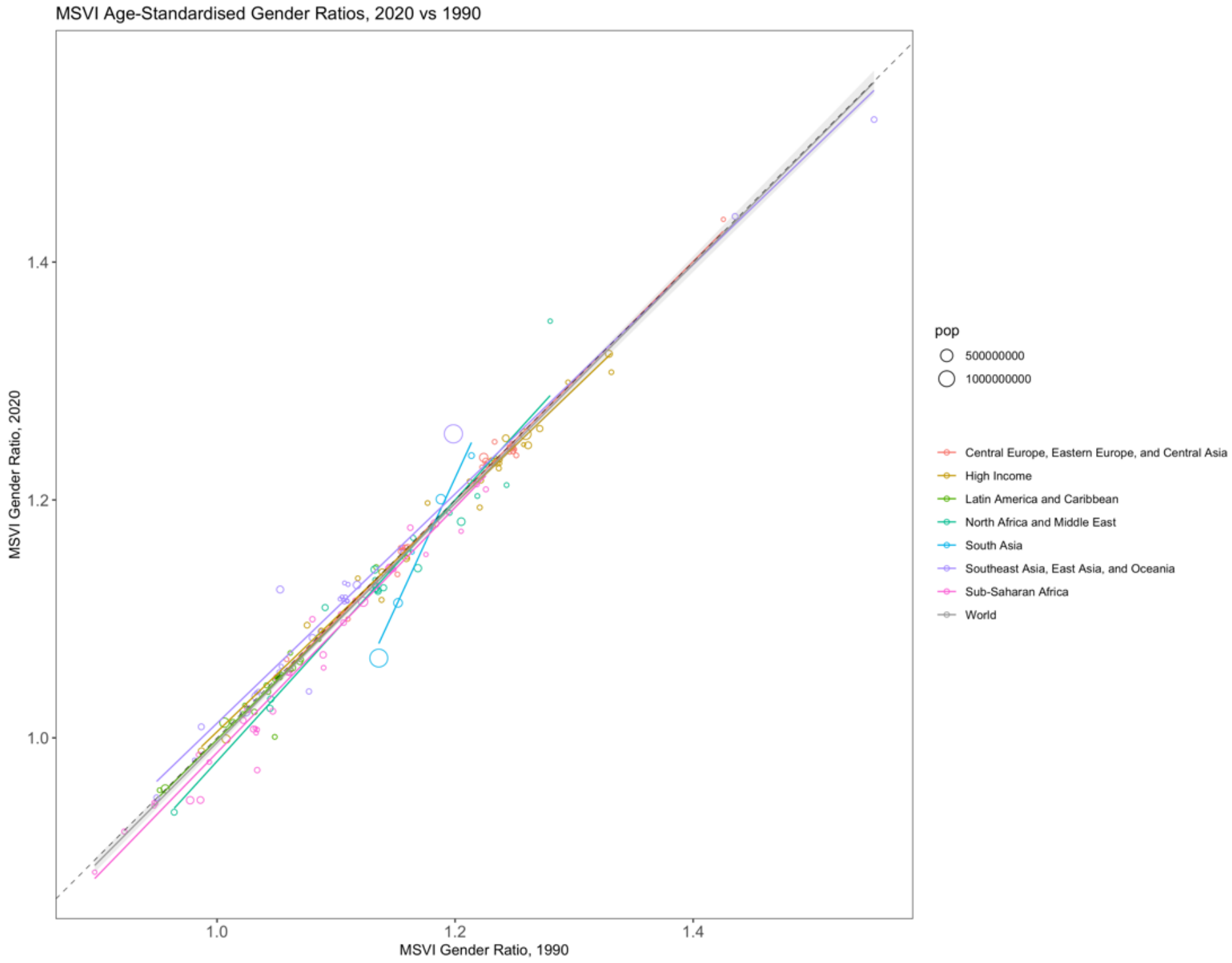

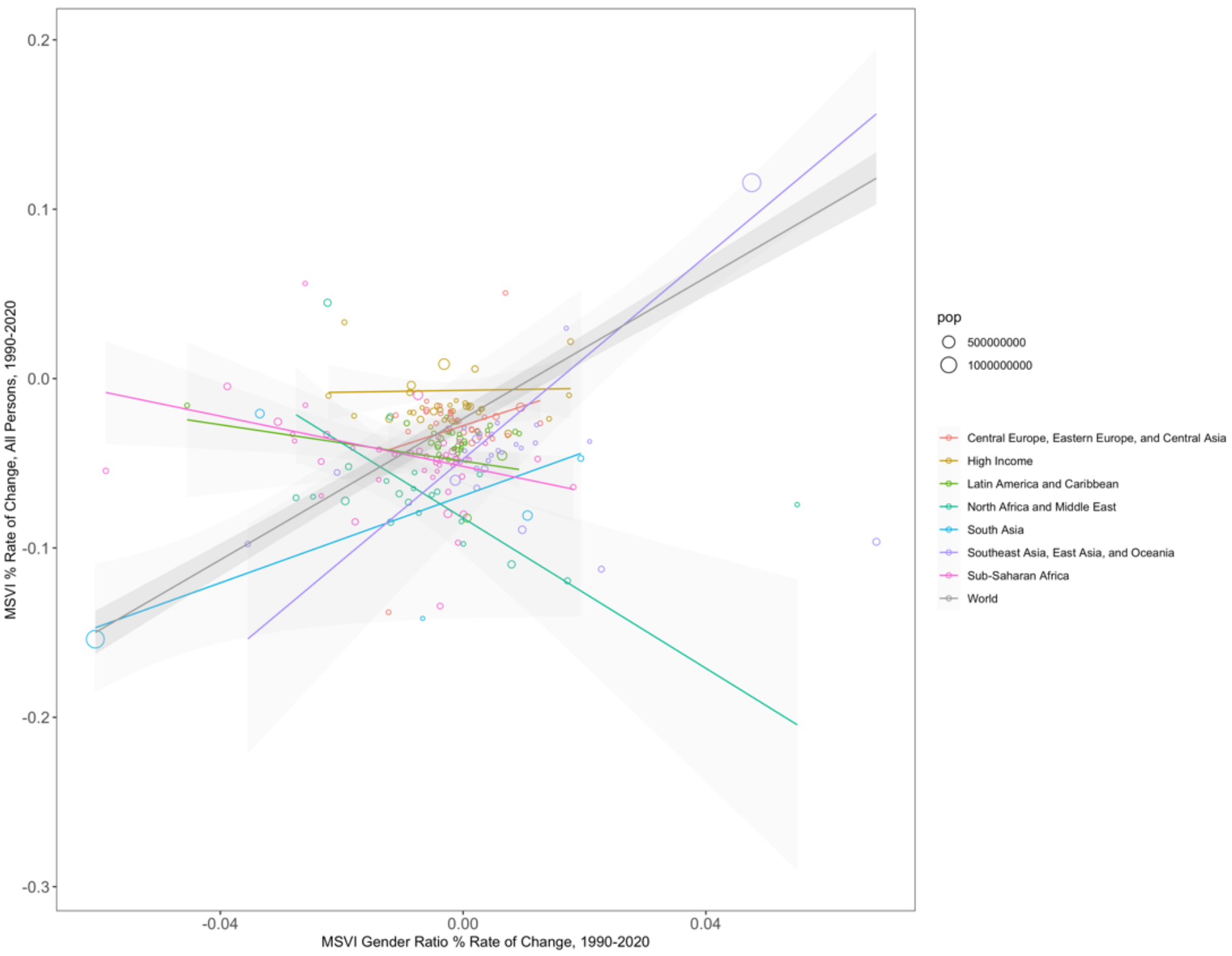

Gray shading refers to the estimate and confidence bands of the global, non-super-regional coefficient
